# Evidence and Characteristics of Traditional Chinese Medicine for Coronary Heart Disease Patients With Anxiety or Depression: A Meta-Analysis and Systematic Review

**DOI:** 10.3389/fphar.2022.854292

**Published:** 2022-05-05

**Authors:** Baofu Wang, Yu Teng, Yang Li, Sijia Lai, Yang Wu, Shiqi Chen, Tong Li, Xiaowan Han, Hufang Zhou, Yu Wang, Ziwen Lu, Haiyan Li, Yukun Ding, Liang Ma, Mingjing Zhao, Xian Wang

**Affiliations:** ^1^ Dongzhimen Hospital, Beijing University of Chinese Medicine, Beijing, China; ^2^ The First Hospital of Hebei Medical University, Shijiazhuang, China; ^3^ Institute of Cardiovascular Diseases, Beijing University of Chinese Medicine, Beijing, China

**Keywords:** coronary heart disease, anxiety, depression, Chinese herbal medicine, efficacy

## Abstract

**Aims:** The objective of this study was to assess the efficacy and potential mechanisms of Chinese herbal medicine (CHM) for treating coronary heart disease (CHD) patients with anxiety or depression.

**Methods:** A systematic literature search was performed. Screening studies, extracting data, and assessing article quality were carried out independently by two researchers. The active ingredients of CHM for the treatment of CHD with anxiety or depression were analyzed by the network pharmacology, and the main potential mechanisms were summarized by the database of Web of Science.

**Results:** A total of 32 studies were included. The results showed that compared with the blank control groups, CHM was more beneficial in treating anxiety or depression in patients with CHD [anxiety: OR = 3.22, 95% CI (1.94, 5.35), *p* < 0.00001, I^2^ = 0%; depression: OR = 3.27, 95% CI (1.67, 6.40), *p* = 0.0005, I^2^ = 0%], and the efficacy of CHM was not inferior to that of Western medicine (WM) [anxiety: OR = 1.58, 95%CI (0.39, 6.35), *p* = 0.52, I^2^ = 67%; depression: OR = 1.97, 95%CI (0.73, 5.28), *p* = 0.18, I^2^ = 33%,]. Additionally, CHM also showed a significant advantage in improving angina stability (AS) in CHD patients with anxiety or depression compared with blank groups [anxiety: SMD = 0.55, 95%CI (0.32, 0.79), *p* < 0.00001, I^2^ = 0%; depression: *p* = 0.004] and WM groups [anxiety: SMD = 1.14, 95%CI (0.80, 1.47), *p* < 0.00001, I^2^ = 0%; depression: SMD = 12.15, 95%CI (6.07, 18.23), *p* < 0.0001, I^2^ = 0%]. Angina frequency (AF) and electrocardiogram (ECG) analysis after using CHM demonstrated similar trends. Based on the network pharmacology, quercetin, kaempferol, luteolin, beta-sitosterol, puerarin, stigmasterol, isorhamnetin, baicalein, tanshinone IIa, and nobiletin were most closely and simultaneously related to the pathological targets of CHD, anxiety, and depression. The main underlying mechanisms might involve anti-damage/apoptosis, anti-inflammation, antioxidative stress, and maintaining neurotransmitter homeostasis.

**Conclusion:** CHM exhibited an obvious efficacy in treating CHD patients with anxiety or depression, especially for improving the symptom of angina pectoris. The most active compounds of CHM could simultaneously act on the pathological targets of CHD, anxiety, and depression. Multiple effective components and multiple targets were the advantages of CHM compared with WM.

## Introduction

Anxiety and depression are commonly found in patients with coronary heart disease (CHD), and the prevalence of CHD complicated with anxiety or depression is 21 and 13%, respectively (Daniel et al., 2018). Percutaneous coronary intervention (PCI) treatment increases the prevalence of anxiety and depression symptoms in CHD patients (Gu et al., 2016). Accumulating evidence has demonstrated that anxiety and depression are associated with the increased risk of CHD (Roest et al., 2010; Lederbogen and Ströhle, 2012; Giannarelli et al., 2017), and the use of anxiolytics or antidepressants is necessary for CHD patients with anxiety or depression. However, current drugs for emotional disorders, such as serotonin-specific reuptake inhibitors (SSRIs) and benzodiazepines, usually exert their effects after several weeks of treatment, with some unwanted side effects (Lakhan and Vieira, 2010; Ko et al., 2020). Thus, a more optimized treatment option is needed.

As an important treatment strategy, Chinese herbal medicine (CHM) is characterized by multiple components, multiple targets, and multiple channels. It has been verified that CHM had a satisfactory efficacy and fewer adverse effects on CHD with anxiety or depression (Liu and Qin, 2016; Ma et al., 2019). However, due to poor methodological quality and limited sample size, the evidence to support the effect of CHM on CHD with anxiety or depression is still weak. Moreover, the possible underlying mechanisms *via* which CHM treats CHD patients with anxiety or depression is still needed to be clarified. Therefore, by comprehensively analyzing published studies, a meta-analysis and systematic review were performed to assess the efficacy of CHM and the underlying mechanisms in the treatment of CHD patients with anxiety or depression, which might provide an essential clinical value for the disease management in the future.

## Methods

This meta-analysis and systematic review were performed in accordance with the Preferred Reporting Items for Systematic Reviews and Meta-Analyses Guidelines (Moher et al., 2009).

### Information Source and Search Strategy

Published articles were searched comprehensively in electronic databases (PubMed, Web of Science, Embase, Cochrane, China National Knowledge Infrastructure, WanFang Data, VIP Database, and SinoMed) up to October 2021. “Coronary disease OR coronary artery disease OR myocardial infarction OR acute coronary syndrome” AND “anxiety OR depression OR depressive disorder” AND “traditional Chinese medicine OR herbal medicine” AND “randomized controlled trial” and their common synonyms were used for the searching strategy. The detailed searching strategy was shown in supplementary material.

### Inclusion and Exclusion Criteria

The inclusion criteria of the articles were as follows: 1) all participants met the diagnostic criteria of CHD with anxiety or depression; 2) the number of subjects in each group was not less than 30; 3) CHD patients in control and trial groups received basic treatments, with antianxiety or antidepressant Western medicine (WM) used (WM groups) or not (blank control groups) in control groups, and oral CHM was used in trial groups; 4) Hamilton anxiety scale (HAMA) and Hamilton depression scale (HAMD) were used to evaluate patients’ anxiety and depression, respectively; and 5) the efficacy index of CHD included one of the following: ① electrocardiogram (ECG); ② angina stability (AS) and angina frequency (AF) come from Seattle Angina Questionnaire; and ③ traditional Chinese medicine syndrome (TCMS) score.

The exclusion criteria of the studies were as follows: 1) Nonclinical study and irrelevant research; 2) CHM was used in control groups, antianxiety or antidepressant WM was used in trial groups; 3) articles with incomplete data; 4) articles more than one high-risk item; and 5) review, meta-analysis, and conference abstracts.

### Study Selection and Data Extraction

Retrieved articles were assessed independently by two researchers (YT and SL) according to the inclusion and exclusion criteria. Data, including first authors’ name, year of publication, sample size, age, gender, diseases, therapeutic regimen, final duration of treatment, dosage form and compositions of TCM, and the outcome index, were extracted from the included studies. The CHM was reported in scientific name, not the Latin name in pharmacopeia to avoid confusion (Table 1) (Rivera et al., 2014). Any disagreements were resolved by discussing and consulting with corresponding authors (MZ and XW).

**TABLE 1:** Compositions of formulation and patented drugs.

Study (year)	Formulation or patented drugs	Source	Compositions	Quality control reported?	Chemical analysis reported?
Anxiety					
Mo 2016 Mo et al. (2016)	Wuling capsule	Zhejiang Zoli Pharmaceutical Company	*Xylaria nigripes* (KL.) Sacc [Xylaria; Wuling mycelia]	Y—Prepared according to NMPA:Z199900—18	N
Guo 2017 Guo (2017)	Shenchai Shuxin decoction	Pharmacy of The First Affiliated Hospital of Heilongjiang University of TCM	*Salvia miltiorrhiza* Bunge [Lamiaceae; Salviae miltiorrhizae radix et rhizoma] 15 g, *Bupleurum chinense* DC. [Apiaceae; Bupleuri radix] 15 g, *Ligusticum chuanxiong* Hort. [Apiaceae; Chuanxiong rhizoma] 15 g, *Corydalis yanhusuo* (Y.H.Chou & Chun C.Hsu) W.T.Wang ex Z.Y.Su and C.Y.Wu [Papaveraceae; Corydalis rhizoma] 15 g, *Platycodon grandiflorus* (Jacq.) A.DC. [Campanulaceae; Platycodonis radix] 10 g, *Curcuma aromatica* Salisb. [Zingiberaceae; Curcumae radix] 10 g, *Citrus × aurantium* L. [Rutaceae; Aurantii fructus] 10 g, *Paeonia lactiflora* Pall. [Paeoniaceae; Paeoniae radix alba] 20 g, *Albizia julibrissin* Durazz. [Fabaceae; Albiziae cortex] 15 g, *Lilium lancifolium* Thunb. [Liliaceae; Lilii bulbus] 20 g, *Reynoutria multiflora* (Thunb.) Moldenke [Polygonaceae; Polygoni multiflori caulis] 20 g, *Glycyrrhiza uralensis* Fisch. ex DC. [Fabaceae; Glycyrrhizae radix et rhizoma praeparata cum melle] 10 g	N	N
Li 2017 Li G. Y. et al. (2017)	Tiaogan Jianpi decoction	—	*Bupleurum chinense* DC. [Apiaceae; Bupleuri radix] 10 g, *Paeonia anomala* subsp. veitchii (Lynch) D.Y.Hong and K.Y.Pan [Paeoniaceae; Paeoniae radix rubra] 10 g, *Atractylodes macrocephala* Koidz. [Asteraceae; Atractylodis macrocephalae rhizoma] 20 g, *Poria cocos* (Schw.)Wolf [Polyporaceae; Poria] 20 g, *Pseudostellaria heterophylla* (Miq.) Pax [Caryophyllaceae; Pseudostellariae radix] 15 g, *Neolitsea cassia* (L.) Kosterm. [Lauraceae; Cinnamomi ramulus] 10 g, *Allium chinense* G.Don [Amaryllidaceae; Allii macrostemonis bulbus] 10 g, *Ligusticum chuanxiong* Hort. [Apiaceae; Chuanxiong rhizoma] 20 g, *Citrus × aurantium* L. [Rutaceae; Aurantii fructus] 10 g, *Corydalis yanhusuo* (Y.H.Chou & Chun C.Hsu) W.T.Wang ex Z.Y.Su and C.Y.Wu [Papaveraceae; Corydalis rhizoma] 10 g, *Pheretima aspergillum* (E. Perrier) [Megascolecidae; Pheretima] 10 g, *Schisandra chinensis* (Turcz.) Baill. [Schisandraceae; Schisandrae chinensis fructus] 5 g, *Mentha canadensis* L. [Lamiaceae; Menthae haplocalycis herba] 10 g, *Gardenia jasminoides* J.Ellis [Rubiaceae; Gardeniae fructus] 5 g, *Glycyrrhiza uralensis* Fisch. ex DC. [Fabaceae; Glycyrrhizae radix et rhizoma praeparata cum melle] 10 g	N	N
Qi 2017 Qi and Song (2017)	Jieyu Tongmai granule	—	*Astragalus mongholicus* Bunge [Fabaceae; Astragali radix], *Angelica sinensis* (Oliv.) Diels [Apiaceae; Angelicae sinensis radix], *Paeonia lactiflora* Pall. [Paeoniaceae; Paeoniae radix alba], *Bupleurum chinense *DC. [Apiaceae; Bupleuri radix], *Lilium lancifolium* Thunb. [Liliaceae; Lilii bulbus], *Gardenia jasminoides* J.Ellis [Rubiaceae; Gardeniae fructus], *Poria cocos* (Schw.)Wolf [Polyporaceae; Poria], *Atractylodes macrocephala* Koidz. [Asteraceae; Atractylodis macrocephalae rhizoma], *Glycyrrhiza uralensis* Fisch. ex DC. [Fabaceae; Glycyrrhizae radix et rhizoma], *Citrus × aurantium* L. [Rutaceae; Aurantii fructus], *Citrus × aurantium* L. [Rutaceae; Citri reticulatae pericarpium viride]	N	N
Zhang 2017 Zhang et al. (2017)	Wuling capsule	Zhejiang Zoli Pharmaceutical Company	*Xylaria nigripes* (KL.) Sacc [Xylaria; Wuling mycelia]	Y—Prepared according to NMPA:Z199900—18	N
Qin 2018 Qin (2018)	Yuxin decoction	—	*Codonopsis pilosula* (Franch.) Nannf. [Campanulaceae; Codonopsis radix] 20 g, *Neolitsea cassia* (L.) Kosterm. [Lauraceae; Cinnamomi ramulus] 12 g, *Panax notoginseng* (Burkill) F.H.Chen [Araliaceae; Notoginseng radix et rhizoma] 12 g, *Bupleurum chinense* DC. [Apiaceae; Bupleuri radix] 15 g, *Citrus × aurantium* L. [Rutaceae; Aurantii fructus] 12 g, *Ziziphus jujuba* Mill. [Rhamnaceae; Ziziphi spinosae semen] 12 g, *Crataegus pinnatifida* Bunge [Rosaceae; Crataegi fructus] 12 g, *Trichosanthes kirilowii* Maxim. [Cucurbitaceae; Trichosanthis pericarpium] 12 g, *Glycyrrhiza uralensis* Fisch. ex DC. [Fabaceae; Glycyrrhizae radix et rhizoma praeparata cum melle] 9 g	N	N
Wang 2018 Wang C. (2018)	Chaihu Longgu Muli granule	Beijing Kangrentang Pharmaceutical Co. LTD.	*Bupleurum chinense* DC. [Apiaceae; Bupleuri radix] 12 g, Os Draconis 15 g, *Ostrea gigas* Thunberg [Ostreidae; Ostreae concha] 15 g, *Scutellaria baicalensis* Georgi [Lamiaceae; Scutellariae radix] 9 g, *Zingiber officinale* Roscoe [Zingiberaceae; Zingiberis rhizoma recens] 9 g, *Codonopsis pilosula* (Franch.) Nannf. [Campanulaceae; Codonopsis radix] 9 g, *Neolitsea cassia* (L.) Kosterm. [Lauraceae; Cinnamomi ramulus] 9 g, *Poria cocos* (Schw.)Wolf [Polyporaceae; Poria] 15 g, *Pinellia ternata *(Thunb.) Makino [Araceae; Pinelliae rhizoma] 9 g, *Rheum palmatum* L. [Polygonaceae; Rhei radix et rhizoma] 9 g, *Pteria martensii* (Dunker) [Pteriidae; Margarita] 15 g, *Ziziphus jujuba* Mill. [Rhamnaceae; Jujubae fructus] 10 g	N	N
Chen 2019 Chen (2019)	Chaihu Jieyu decoction	—	*Bupleurum chinense* DC. [Apiaceae; Bupleuri radix] 15 g, *Citrus × aurantium* L. [Rutaceae; Citri reticulatae pericarpium] 15 g, *Ligusticum chuanxiong* Hort. [Apiaceae; Chuanxiong rhizoma] 15 g, *Cyperus rotundus* L. [Cyperaceae; Cyperi rhizoma] 15 g, *Citrus × aurantium* L. [Rutaceae; Aurantii fructus] 10 g, *Paeonia lactiflora* Pall. [Paeoniaceae; Paeoniae radix alba] 10 g, *Angelica sinensis* (Oliv.) Diels [Apiaceae; Angelicae sinensis radix] 10 g, *Salvia miltiorrhiza* Bunge [Lamiaceae; Salviae miltiorrhizae radix et rhizoma] 15 g, *Curcuma aromatica* Salisb. [Zingiberaceae; Curcumae radix] 15 g, *Corydalis yanhusuo* (Y.H.Chou & Chun C.Hsu) W.T.Wang ex Z.Y.Su and C.Y.Wu [Papaveraceae; Corydalis rhizoma] 15 g, *Trichosanthes kirilowii* Maxim. [Cucurbitaceae; Trichosanthis fructus] 15 g, *Pinellia ternata* (Thunb.) Makino [Araceae; Pinelliae rhizoma] 15 g	N	N
Dong 2019 Dong (2019)	Danqi Anshen decoction	Heilongjiang University of TCM	*Salvia miltiorrhiza* Bunge [Lamiaceae; Salviae miltiorrhizae radix et rhizoma], *Astragalus mongholicus* Bunge [Fabaceae; Astragali radix], *Angelica sinensis* (Oliv.) Diels [Apiaceae; Angelicae sinensis radix], *Rehmannia glutinosa* (Gaertn.) DC. [Orobanchaceae; Rehmanniae radix praeparata], *Paeonia lactiflora* Pall. [Paeoniaceae; Paeoniae radix alba], *Ligusticum chuanxiong* Hort. [Apiaceae; Chuanxiong rhizoma], *Codonopsis pilosula* (Franch.) Nannf. [Campanulaceae; Codonopsis radix], Os Draconis, *Ostrea gigas* Thunberg [Ostreidae; Ostreae concha], *Bupleurum chinense* DC. [Apiaceae; Bupleuri radix], *Cyperus rotundus* L. [Cyperaceae; Cyperi rhizoma], *Albizia julibrissin* Durazz. [Fabaceae; Albiziae flos], *Glycyrrhiza uralensis* Fisch. ex DC. [Fabaceae; Glycyrrhizae radix et rhizoma]	N	N
Yang 2019 Yang (2019)	Chaihu Longgu Muli granule	Beijing Kangrentang Pharmaceutical Co. LTD.	*Bupleurum chinens*e DC. [Apiaceae; Bupleuri radix] 10 g, Os Draconis 15 g, *Ostrea gigas* Thunberg [Ostreidae; Ostreae concha] 15 g, *Scutellaria baicalensis* Georgi [Lamiaceae; Scutellariae radix] 10 g, *Zingiber officinale* Roscoe [Zingiberaceae; Zingiberis rhizoma recens] 10 g, *Codonopsis pilosula* (Franch.) Nannf. [Campanulaceae; Codonopsis radix] 10 g, *Neolitsea cassia* (L.) Kosterm. [Lauraceae; Cinnamomi ramulus] 10 g, *Poria cocos* (Schw.)Wolf [Polyporaceae; Poria] 15 g, *Pinellia ternata* (Thunb.) Makino [Araceae; Pinelliae rhizoma] 10 g, *Rheum palmatum* L. [Polygonaceae; Rhei radix et rhizoma] 10 g, *Pteria martensii* (Dunker) [Pteriidae; Margarita] 15 g, *Ziziphus jujuba* Mill. [Rhamnaceae; Jujubae fructus] 10 g	N	N
Zhang 2019 Zhang (2019)	Jiangqi Dayu decoction	Pharmacy of Affiliated Hospital of Liaoning University of TCM	*Bupleurum chinense* DC. [Apiaceae; Bupleuri radix] 15 g, *Dolomiaea costus* (Falc.) Kasana and A.K.Pandey [Asteraceae; aucklandiae radix] 15 g, *Citrus × aurantium* L. [Rutaceae; Aurantii fructus] 15 g, *Platycodon grandiflorus* (Jacq.) A.DC. [Campanulaceae; Platycodonis radix] 15 g, *Cyperus rotundus* L. [Cyperaceae; Cyperi rhizoma] 15 g, *Curcuma aromatica* Salisb. [Zingiberaceae; Curcumae radix] 15 g, *Albizia julibrissin* Durazz. [Fabaceae; Albiziae flos] 15 g, *Reynoutria multiflora* (Thunb.) Moldenke [Polygonaceae; Polygoni multiflori caulis] 20 g, *Glycyrrhiza uralensis* Fisch. ex DC. [Fabaceae; Glycyrrhizae radix et rhizoma praeparata cum melle] 10 g	N	N
Zhao 2019 Zhao et al. (2019)	Xinling pill	—	*Selenarctos thibetanus* G. Cuvier [Ursidae; Ursi fellis pulvis], *Moschus berezovskii* Flerov [Cervidae; Moschus], *Bos taurus domesticus* Gemlin [Bovidae; Bovis calculus], *Pteria martensii* (Dunker) [Pteriidae; Margarita], *Panax ginseng* C.A.Mey. [Araliaceae; Ginseng radix et rhizoma], *Panax notoginseng* (Burkill) F.H.Chen [Araliaceae; Notoginseng radix et rhizoma], *Dryobalanops aromatica* C.F.Gaertn. [Dipterocarpaceae; Borneolum syntheticum], *Bufo bufo gargarizans* Cantor [Bufonidae; Bufonis venenum], *Bubalus bubalis* Linnaeus [Bovidae; Cornu bubali]	N	N
Jin 2021 Jin et al. (2021)	Shuxin decoction	Pharmacy of Dalian Municipal Hospital and The Second Affiliated Hospital of Liaoning University of TCM	*Bupleurum chinense* DC. [Apiaceae; Bupleuri radix] 15 g, *Glycyrrhiza uralensis* Fisch. ex DC. [Fabaceae; Glycyrrhizae radix et rhizoma praeparata cum melle] 20 g, *Triticum aestivum* L. [Poaceae; *Triticum aestivum*] 100 g, *Poria cocos* (Schw.) Wolf [Polyporaceae; Poria] 20 g, *Citrus × aurantium* L. [Rutaceae; Citri reticulatae pericarpium] 15 g, *Pinellia ternata *(Thunb.) Makino [Araceae; Pinelliae rhizoma] 15 g, *Citrus × aurantium* L. [Rutaceae; Aurantii fructus immaturus] 15 g, *Allium chinense* G.Don [Amaryllidaceae; Allii macrostemonis bulbus] 15 g, *Trichosanthes kirilowii* Maxim. [Cucurbitaceae; Trichosanthis fructus] 20 g, *Ziziphus jujuba* Mill. [Rhamnaceae; Jujubae fructus] 15 g, *Zingiber officinale* Roscoe [Zingiberaceae; Zingiberis rhizoma recens] 10 g	N	N
Wang 2021 Wang et al. (2021)	Shuxin oral liquid	Hubei Minkang Pharmaceutical Co. LTD.	*Codonopsis pilosula* (Franch.) Nannf. [Campanulaceae; Codonopsis radix], *Astragalus mongholicus* Bunge [Fabaceae; Astragali radix], *Carthamus tinctorius* L. [Asteraceae; Carthami flos], *Angelica sinensis* (Oliv.) Diels [Apiaceae; Angelicae sinensis radix], *Ligusticum chuanxiong* Hort. [Apiaceae; Chuanxiong rhizoma], *Sparganium stoloniferum* (Buch.-Ham. ex Graebn.) Buch.-Ham. ex Juz. [Typhaceae; Sparganii rhizoma], *Typha angustifolia* L. [Typhaceae; Typhae pollen]	Y—Prepared according to NMPA:Z10900011	N
Zhang 2021 Zhang and Jin (2021)	Xuefu Zhuyu decoction and Yueju pill	—	*Prunus persica* (L.) Batsch [Rosaceae; Persicae semen] 12 g, *Carthamus tinctorius* L. [Asteraceae; Carthami flos] 9 g, *Angelica sinensis* (Oliv.) Diels [Apiaceae; Angelicae sinensis radix] 9 g, *Paeonia anomala* subsp. veitchii (Lynch) D.Y.Hong and K.Y.Pan [Paeoniaceae; Paeoniae radix rubra] 9 g, *Ligusticum chuanxiong* Hort. [Apiaceae; Chuanxiong rhizoma] 6 g, *Rehmannia glutinosa* (Gaertn.) DC. [Orobanchaceae; Rehmanniae radix] 9 g, *Citrus × aurantium* L. [Rutaceae; Aurantii fructus] 6 g, *Cyathula officinalis* K.C.Kuan [Amaranthaceae; Cyathulae radix] 9 g, *Bupleurum chinense* DC. [Apiaceae; Bupleuri radix] 6 g, *Platycodon grandiflorus* (Jacq.) A.DC. [Campanulaceae; Platycodonis radix] 6 g, *Glycyrrhiza uralensis* Fisch. ex DC. [Fabaceae; Glycyrrhizae radix et rhizoma praeparata cum melle] 6 g, *Cyperus rotundus* L. [Cyperaceae; Cyperi rhizoma] 15 g, *Gardenia jasminoides* J.Ellis [Rubiaceae; Gardeniae fructus] 15 g, Massa Medicata Fermentata 10 g	N	N
Depression					
Sun 2011 Sun (2011)	Jieyu Anshen decoction	—	*Bupleurum chinense* DC. [Apiaceae; Bupleuri radix] 12 g, *Paeonia lactiflora* Pall. [Paeoniaceae; Paeoniae radix alba] 30 g, *Cyperus rotundus* L. [Cyperaceae; Cyperi rhizoma] 12 g, *Citrus × aurantium* L. [Rutaceae; Aurantii fructus] 12 g, *Curcuma aromatica* Salisb. [Zingiberaceae; Curcumae radix] 12 g, *Albizia julibrissin* Durazz. [Fabaceae; Albiziae flos] 12 g, *Angelica sinensis* (Oliv.) Diels [Apiaceae; Angelicae sinensis radix] 12 g, *Ligusticum chuanxiong* Hort. [Apiaceae; Chuanxiong rhizoma] 12 g, *Ziziphus jujuba* Mill. [Rhamnaceae; Ziziphi spinosae semen] 30 g, *Citrus × aurantium* L. [Rutaceae; Citri reticulatae pericarpium] 12 g, *Wurfbainia villosa* (Lour.) Skornick. and A.D.Poulsen [Zingiberaceae; Amomi fructus] 6 g, *Glycyrrhiza uralensis* Fisch. ex DC. [Fabaceae; Glycyrrhizae radix et rhizoma] 6 g	N	N
Lin 2012 Lin (2012)	Xiaoyao pill	Lanzhou Taibao Pharmaceutical Co. LTD.	*Angelica sinensis* (Oliv.) Diels [Apiaceae; Angelicae sinensis radix], *Paeonia lactiflora* Pall. [Paeoniaceae; Paeoniae radix alba], *Bupleurum chinense* DC. [Apiaceae; Bupleuri radix], *Poria cocos* (Schw.)Wolf [Polyporaceae; Poria], *Atractylodes macrocephala* Koidz. [Asteraceae; Atractylodis macrocephalae rhizoma], *Zingiber officinale *Roscoe [Zingiberaceae; Zingiberis rhizoma recens], *Glycyrrhiza uralensis* Fisch. ex DC. [Fabaceae; Glycyrrhizae radix et rhizoma], *Mentha canadensis* L. [Lamiaceae; Menthae haplocalycis herba]	Y—Prepared according to NMPA: Z62021225	N
Zhang 2012 Zhang et al. (2012)	Jiawei Shengdan Louxie Sini granule	Pharmacy of Xiyuan Hospital, China Academy of Chinese Medical Sciences	*Codonopsis pilosula* (Franch.) Nannf. [Campanulaceae; Codonopsis radix] 12 g, *Ophiopogon japonicus* (Thunb.) Ker Gawl. [Asparagaceae; Ophiopogonis radix] 9 g, *Schisandra chinensis *(Turcz.) Baill. [Schisandraceae; Schisandrae chinensis fructus] 6 g, *Salvia miltiorrhiza* Bunge [Lamiaceae; Salviae miltiorrhizae radix et rhizoma] 30 g, *Santalum album* L. [Santalaceae; Santali albi lignum] 9 g, *Wurfbainia villosa* (Lour.) Skornick. and A.D.Poulsen [Zingiberaceae; Amomi fructus] 3 g, *Trichosanthes kirilowii* Maxim. [Cucurbitaceae; Trichosanthis fructus] 12 g, *Allium chinense* G.Don [Amaryllidaceae; Allii macrostemonis bulbus] 10 g, *Pinellia ternata *(Thunb.) Makino [Araceae; Pinelliae rhizoma] 9 g, *Bupleurum chinense* DC. [Apiaceae; Bupleuri radix] 12 g, *Citrus × aurantium* L. [Rutaceae; Aurantii fructus] 9 g, *Paeonia anomala* subsp. veitchii (Lynch) D.Y.Hong and K.Y.Pan [Paeoniaceae; Paeoniae radix rubra] 9 g, *Glycyrrhiza uralensis* Fisch. ex DC. [Fabaceae; Glycyrrhizae radix et rhizoma] 6 g, *Angelica sinensis* (Oliv.) Diels [Apiaceae; Angelicae sinensis radix] 9 g, *Lycium barbarum* L. [Solanaceae; Lycii fructus] 12 g, *Cuscuta chinensis* Lam. [Convolvulaceae; Cuscutae semen] 12 g	N	N
Qin 2013 Qin and Liu (2013)	Tongxin Jieyu granule	Pharmacy of Longhua Hospital affiliated to Shanghai University of TCM	*Astragalus mongholicus* Bunge [Fabaceae; Astragali radix] 30 g, *Trichosanthes kirilowii* Maxim. [Cucurbitaceae; Trichosanthis fructus] 15 g, *Salvia miltiorrhiza *Bunge [Lamiaceae; Salviae miltiorrhizae radix et rhizoma] 12 g, *Bupleurum chinense* DC. [Apiaceae; Bupleuri radix] 15 g, *Corydalis yanhusuo *(Y.H.Chou & Chun C.Hsu) W.T.Wang ex Z.Y.Su and C.Y.Wu [Papaveraceae; Corydalis rhizoma] 15 g, *Poria cocos* (Schw.)Wolf [Polyporaceae; Poria] 20 g, *Curcuma aromatica *Salisb. [Zingiberaceae; Curcumae radix] 15 g, *Citrus medica *L. [Rutaceae; Citri sarcodactylis fructus] 10 g, *Dolomiaea costus* (Falc.) Kasana and A.K.Pandey [Asteraceae; aucklandiae radix] 10 g	N	N
Zhu 2013 Zhu (2013)	Jieyu granule	Weifang Hospital of TCM	*Bupleurum chinense* DC. [Apiaceae; Bupleuri radix], *Melia azedarach* L. [Meliaceae; Toosendan fructus], *Citrus × aurantium* L. [Rutaceae; Citri reticulatae pericarpium viride], *Paeonia lactiflora* Pall. [Paeoniaceae; Paeoniae radix alba], *Citrus medica* L. [Rutaceae; Citri fructus], *Dalbergia odorifera* T.C.Chen [Fabaceae; Dalbergiae odoriferae lignum], *Gardenia jasminoides* J.Ellis [Rubiaceae; Gardeniae fructus], *Scutellaria baicalensis* Georgi [Lamiaceae; Scutellariae radix], *Acorus calamus* var. angustatus Besser [Acoraceae; Acori tatarinowii rhizoma], *Albizia julibrissin* Durazz. [Fabaceae; Albiziae cortex], *Ziziphus jujuba* Mill. [Rhamnaceae; Ziziphi spinosae semen], succinum	N	N
Gu 2014 Gu et al. (2014)	Shugan Jieyu decoction	—	*Bupleurum chinense* DC. [Apiaceae; Bupleuri radix] 15 g, *Ligusticum chuanxiong* Hort. [Apiaceae; Chuanxiong rhizoma] 10 g, *Cyperus rotundus* L. [Cyperaceae; Cyperi rhizoma] 10 g, *Citrus × aurantium* L. [Rutaceae; Aurantii fructus] 12 g, *Curcuma aromatica* Salisb. [Zingiberaceae; Curcumae radix] 12 g, *Corydalis yanhusuo *(Y.H.Chou & Chun C.Hsu) W.T.Wang ex Z.Y.Su and C.Y.Wu [Papaveraceae; Corydalis rhizoma] 15 g, *Paeonia lactiflora* Pall. [Paeoniaceae; Paeoniae radix alba] 12 g, *Glycyrrhiza uralensis* Fisch. ex DC. [Fabaceae; Glycyrrhizae radix et rhizoma] 6 g	N	N
Shang 2014 Shang et al. (2014)	Chaihu Longgu Muli decoction	—	*Bupleurum chinense* DC. [Apiaceae; Bupleuri radix] 10 g, *Astragalus mongholicus* Bunge [Fabaceae; Astragali radix] 30 g, *Scutellaria baicalensis* Georgi [Lamiaceae; Scutellariae radix] 10 g, *Pinellia ternata* (Thunb.) Makino [Araceae; Pinelliae rhizoma praeparatum cum alumine] 10 g, *Zingiber officinale* Roscoe [Zingiberaceae; Zingiberis rhizoma recens] 10 g, *Neolitsea cassia *(L.) Kosterm. [Lauraceae; Cinnamomi ramulus] 10 g, *Poria cocos* (Schw.)Wolf [Polyporaceae; Poria] 20 g, Magnetitum 10 g, *Rheum palmatum* L. [Polygonaceae; Rhei radix et rhizoma] 10 g, *Gardenia jasminoides* J.Ellis [Rubiaceae; Gardeniae fructus] 10 g, *Polygala tenuifolia* Willd. [Polygalaceae; Polygalae radix] 10 g, *Forsythia suspensa *(Thunb.) Vahl [Oleaceae; Forsythiae fructus] 10 g, *Ziziphus jujuba* Mill. [Rhamnaceae; Jujubae fructus] 3 pieces, *Citrus × aurantium* L. [Rutaceae; Citri reticulatae pericarpium] 10 g, Os Draconis 30 g, *Ostrea gigas* Thunberg [Ostreidae; Ostreae concha] 30 g	N	N
Mu 2015 Mu (2015)	Yangxin Jieyu decoction	Pharmacy of Shandong Hospital of TCM	*Astragalus mongholicus* Bunge [Fabaceae; Astragali radix] 15 g, *Codonopsis pilosula* (Franch.) Nannf. [Campanulaceae; Codonopsis radix] 15 g, *Angelica sinensis* (Oliv.) Diels [Apiaceae; Angelicae sinensis radix] 12 g, *Ligusticum chuanxiong* Hort. [Apiaceae; Chuanxiong rhizoma] 9 g, *Bupleurum chinense *DC. [Apiaceae; Bupleuri radix] 9 g, *Paeonia lactiflora* Pall. [Paeoniaceae; Paeoniae radix alba] 12 g, *Salvia miltiorrhiza *Bunge [Lamiaceae; Salviae miltiorrhizae radix et rhizoma] 12 g, *Glycyrrhiza uralensis* Fisch. ex DC. [Fabaceae; Glycyrrhizae radix et rhizoma] 3 g, *Polygala tenuifolia* Willd. [Polygalaceae; Polygalae radix] 6 g, *Schisandra chinensis* (Turcz.) Baill. [Schisandraceae; Schisandrae chinensis fructus] 9 g, *Curcuma aromatica* Salisb. [Zingiberaceae; Curcumae radix] 12 g, *Albizia julibrissin *Durazz. [Fabaceae; Albiziae flos] 12 g, *Ziziphus jujuba* Mill. [Rhamnaceae; Ziziphi spinosae semen] 30 g, *Citrus × aurantium* L. [Rutaceae; Citri reticulatae pericarpium] 9 g, *Wurfbainia villosa* (Lour.) Skornick. and A.D.Poulsen [Zingiberaceae; Amomi fructus] 6 g	N	N
Shi 2016 Shi et al. (2016)	Jieyu Tongmai decoction	Pharmacy of the First Affiliated Hospital of Tianjin University of Chinese Medicine	*Trichosanthes kirilowii* Maxim. [Cucurbitaceae; Trichosanthis fructus] 30 g, *Allium chinense* G.Don [Amaryllidaceae; Allii macrostemonis bulbus] 10 g, *Pinellia ternata* (Thunb.) Makino [Araceae; Pinelliae rhizoma] 10 g, *Angelica sinensis* (Oliv.) Diels [Apiaceae; Angelicae sinensis radix] 10 g, *Dalbergia odorifera* T.C.Chen [Fabaceae; Dalbergiae odoriferae lignum] 10 g, *Paeonia anomala* subsp. veitchii (Lynch) D.Y.Hong and K.Y.Pan [Paeoniaceae; Paeoniae radix rubra] 10 g, *Salvia miltiorrhiza* Bunge [Lamiaceae; Salviae miltiorrhizae radix et rhizoma] 30 g, *Spatholobus suberectus* Dunn [Fabaceae; Spatholobi caulis] 30 g, *Ligusticum chuanxiong* Hort. [Apiaceae; Chuanxiong rhizoma] 10 g, *Cyperus rotundus *L. [Cyperaceae; Cyperi rhizoma] 10 g, *Ziziphus jujuba *Mill. [Rhamnaceae; Ziziphi spinosae semen] 15 g, *Platycladus orientalis* (L.) Franco [Cupressaceae; Platycladi semen] 15 g, *Lilium lancifolium* Thunb. [Liliaceae; Lilii bulbus] 30 g, *Albizia julibrissin* Durazz. [Fabaceae; Albiziae flos] 15 g, *Albizia julibrissin* Durazz. [Fabaceae; Albiziae cortex] 30 g, *Curcuma aromatica *Salisb. [Zingiberaceae; Curcumae radix] 10 g	N	N
Li 2017 Li F. E. et al. (2017)	Jiawei Wendan decoction	—	*Poria cocos* (Schw.)Wolf [Polyporaceae; Poria] 15 g, *Pinellia ternata *(Thunb.) Makino [Araceae; Pinelliae rhizoma] 10 g, *Glycyrrhiza uralensis* Fisch. ex DC. [Fabaceae; Glycyrrhizae radix et rhizoma praeparata cum melle] 10 g, *Citrus × aurantium* L. [Rutaceae; Aurantii fructus immaturus] 10 g, *Bambusa tuldoides* Munro [Poaceae; Bambusae caulis in taenias] 10 g, *Citrus × aurantium* L. [Rutaceae; Citri reticulatae pericarpium] 10 g, *Zingiber officinale* Roscoe [Zingiberaceae; Zingiberis rhizoma recens] 3 pieces, *Ziziphus jujuba* Mill. [Rhamnaceae; Jujubae fructus] 5 pieces, *Salvia miltiorrhiza* Bunge [Lamiaceae; Salviae miltiorrhizae radix et rhizoma] 24 g, *Curcuma aromatica* Salisb. [Zingiberaceae; Curcumae radix] 10 g, *Albizia julibrissin* Durazz. [Fabaceae; Albiziae cortex] 30 g, *Acorus calamus* var. angustatus Besser [Acoraceae; Acori tatarinowii rhizoma] 10 g, *Polygala tenuifolia* Willd. [Polygalaceae; Polygalae radix] 10 g, *Ziziphus jujuba* Mill. [Rhamnaceae; Ziziphi spinosae semen] 30 g, Os Draconis 30 g, *Ostrea gigas* Thunberg [Ostreidae; Ostreae concha] 30 g	N	N
Su 2017 Su (2017)	Suanzaoren decoction	Pharmacy of Shandong Hospital of TCM	*Ziziphus jujuba* Mill. [Rhamnaceae; Ziziphi spinosae semen] 30 g, *Ligusticum chuanxiong* Hort. [Apiaceae; Chuanxiong rhizoma] 12 g, *Poria cocos* (Schw.)Wolf [Polyporaceae; Poria] 15 g, *Anemarrhena asphodeloides* Bunge [Asparagaceae; Anemarrhenae rhizoma] 12 g, *Pseudostellaria heterophylla* (Miq.) Pax [Caryophyllaceae; Pseudostellariae radix] 15 g, *Ophiopogon japonicus *(Thunb.) Ker Gawl. [Asparagaceae; Ophiopogonis radix] 15 g, *Schisandra chinensis* (Turcz.) Baill. [Schisandraceae; Schisandrae chinensis fructus] 9 g, *Eclipta prostrata* (L.) L. [Asteraceae; Ecliptae herba] 15 g, *Ligustrum lucidum *W.T.Aiton [Oleaceae; Ligustri lucidi fructus] 15 g, *Neolitsea cassia* (L.) Kosterm. [Lauraceae; Cinnamomi ramulus] 9 g, *Glycyrrhiza uralensis* Fisch. ex DC. [Fabaceae; Glycyrrhizae radix et rhizoma] 3 g	N	N
Wang1 2018 Wang D. D. (2018)	Dachaihu decoction	—	*Bupleurum chinense* DC. [Apiaceae; Bupleuri radix] 18 g, *Scutellaria baicalensis* Georgi [Lamiaceae; Scutellariae radix] 12 g, *Pinellia ternata* (Thunb.) Makino [Araceae; Pinelliae rhizoma praeparatum cum alumine] 12 g, *Paeonia lactiflora* Pall. [Paeoniaceae; Paeoniae radix alba] 15 g, *Zingiber officinale* Roscoe [Zingiberaceae; Zingiberis rhizoma recens] 9 g, *Citrus × aurantium* L. [Rutaceae; Aurantii fructus immaturus] 10 g, *Glycyrrhiza uralensis* Fisch. ex DC. [Fabaceae; Glycyrrhizae radix et rhizoma] 6 g, *Ziziphus jujuba* Mill. [Rhamnaceae; Jujubae fructus] 3 pieces, *Rheum palmatum* L. [Polygonaceae; Rhei radix et rhizoma] 3 g, *Salvia miltiorrhiza* Bunge [Lamiaceae; Salviae miltiorrhizae radix et rhizoma] 20 g, *Bombyx mori* Linnaeus [Silkworm pilgrimaging; Bombyx batryticatus] 8 g, *Cryptotympana pustulata* Fabricius [Cicadidae; Cicadae periostracum] 8 g, *Curcuma longa* L. [Zingiberaceae; Curcumae longae rhizoma] 12 g	N	N
Wang2 2018 Wang Y. (2018)	Buxinqi decoction	—	*Salvia miltiorrhiza* Bunge [Lamiaceae; Salviae miltiorrhizae radix et rhizoma], *Poria cocos* (Schw.)Wolf [Polyporaceae; Poria], *Atractylodes macrocephala *Koidz. [Asteraceae; Atractylodis macrocephalae rhizoma], *Astragalus mongholicus* Bunge [Fabaceae; Astragali radix], *Angelica sinensis* (Oliv.) Diels [Apiaceae; Angelicae sinensis radix], *Neolitsea cassia* (L.) Kosterm. [Lauraceae; Cinnamomi ramulus], *Glycyrrhiza uralensis* Fisch. ex DC. [Fabaceae; Glycyrrhizae radix et rhizoma]	Y—Prepared according to 2010 Chinese pharmacopeia	N
Shi 2018 Shi (2018)	Tongmai Sanyu granule	Beijing Kangrentang Pharmaceutical Co. LTD.	*Angelica sinensis *(Oliv.) Diels [Apiaceae; Angelicae sinensis radix] 10 g, *Paeonia anomala* subsp. veitchii (Lynch) D.Y.Hong and K.Y.Pan [Paeoniaceae; Paeoniae radix rubra] 10 g, *Salvia miltiorrhiza* Bunge [Lamiaceae; Salviae miltiorrhizae radix et rhizoma] 10 g, *Carthamus tinctorius* L. [Asteraceae; Carthami flos] 10 g, *Bupleurum chinense* DC. [Apiaceae; Bupleuri radix] 10 g, *Glycyrrhiza uralensis* Fisch. ex DC. [Fabaceae; Glycyrrhizae radix et rhizoma praeparata cum melle] 10 g, *Citrus × aurantium* L. [Rutaceae; Aurantii fructus] 10 g, *Albizia julibrissin* Durazz. [Fabaceae; Albiziae cortex] 15 g, *Paeonia lactiflora* Pall. [Paeoniaceae; Paeoniae radix alba] 15 g, *Corydalis yanhusuo* (Y.H.Chou & Chun C.Hsu) W.T.Wang ex Z.Y.Su and C.Y.Wu [Papaveraceae; Corydalis rhizoma] 15 g, *Wurfbainia villosa* (Lour.) Skornick. and A.D.Poulsen [Zingiberaceae; Amomi fructus] 15 g, *Curcuma aromatica* Salisb. [Zingiberaceae; Curcumae radix] 20 g	N	N
Lu 2019 Lu (2019)	Jieyu Shugan Tongmai decoction	Department of TCM, Shenyang Hospital of TCM	*Angelica sinensis* (Oliv.) Diels [Apiaceae; Angelicae sinensis radix] 10 g, P*aeonia anomala* subsp. veitchii (Lynch) D.Y.Hong and K.Y.Pan [Paeoniaceae; Paeoniae radix rubra] 10 g, *Salvia miltiorrhiza* Bunge [Lamiaceae; Salviae miltiorrhizae radix et rhizoma] 10 g, *Carthamus tinctorius* L. [Asteraceae; Carthami flos] 10 g, *Bupleurum chinense* DC. [Apiaceae; Bupleuri radix] 10 g, *Glycyrrhiza uralensis* Fisch. ex DC. [Fabaceae; Glycyrrhizae radix et rhizoma praeparata cum melle] 10 g, *Citrus × aurantium* L. [Rutaceae; Aurantii fructus] 10 g, *Albizia julibrissin* Durazz. [Fabaceae; Albiziae cortex] 15 g, *Lilium lancifolium* Thunb. [Liliaceae; Lilii bulbus] 15 g, *Paeonia lactiflora* Pall. [Paeoniaceae; Paeoniae radix alba] 15 g, *Corydalis yanhusuo* (Y.H.Chou & Chun C.Hsu) W.T.Wang ex Z.Y.Su and C.Y.Wu [Papaveraceae; Corydalis rhizoma] 15 g, *Wurfbainia villosa* (Lour.) Skornick. and A.D.Poulsen [Zingiberaceae; Amomi fructus] 15 g, *Curcuma aromatica* Salisb. [Zingiberaceae; Curcumae radix] 20 g	N	N
Huang 2020 Huang et al. (2020)	Guanxinning tablet	Chia tai Qing Chun Bao pharmaceutical Co. LTD.	*Salvia miltiorrhiza* Bunge [Lamiaceae; Salviae miltiorrhizae radix et rhizoma], *Ligusticum chuanxiong* Hort. [Apiaceae; Chuanxiong rhizoma]	Y—Prepared according to NMPA: Z20150028	N
Zhang 2020 Zhang et al. (2020)	Huatan Guyu recipe	—	*Cyperus rotundus* L. [Cyperaceae; Cyperi rhizoma] 12 g, *Atractylodes lancea* (Thunb.) DC. [Asteraceae; Atractylodis rhizoma] 10 g, *Gardenia jasminoides* J.Ellis [Rubiaceae; Gardeniae fructus] 10 g, *Ligusticum chuanxiong* Hort. [Apiaceae; Chuanxiong rhizoma] 12 g, *Citrus × aurantium* L. [Rutaceae; Citri reticulatae pericarpium] 10 g, *Curcuma aromatica *Salisb. [Zingiberaceae; Curcumae radix] 10 g, *Pinellia ternata* (Thunb.) Makino [Araceae; Pinelliae rhizoma] 10 g, *Wurfbainia villosa* (Lour.) Skornick. and A.D.Poulsen [Zingiberaceae; Amomi fructus] 3 g, *Prunus persica* (L.) Batsch [Rosaceae; Persicae semen] 10 g, *Carthamus tinctorius* L. [Asteraceae; Carthami flos] 10 g	N	N

Note: Co. LTD.: company limited; NMPA: China of National Medical Products Administration; N: NO; Y: Yes.

### Study Quality Assessment

Two authors (YL and YT) independently assessed the methodological quality according to the Cochrane risk-of-bias tool (Zhang K. J. et al., 2019). Sufficient domain information in relevant studies was considered as low risk, inadequate information was regarded as unclear risk, and no related information was regarded as high risk.

### Data Analysis and Synthesis

RevMan 5.3 software provided by the Cochrane Collaboration was used for meta-analysis. The odds ratio (OR) and standard mean difference (SMD) were used to analyze the pooled effects of dichotomous outcomes and continuous variable, respectively. When the heterogeneity of included studies was low (I^2^ < 50%), the fixed effect model was selected to analyze the data; otherwise, a random-effects model was applied. The subgroups analysis was based on whether control groups used WM or not. Sensitivity analysis was performed to explore potential effect modification. Also, funnel plots were used to assess publication bias. *p* < 0.05 was considered statistically significant.

### Chinese Herbal Medicine Compositions and Potential Mechanisms

The frequency statistics of single CHM was performed to identify the commonly used drugs, and CHM with frequency not less than three were selected for network pharmacology to find the primary active ingredients and the disease targets. The targets of the active ingredient of CHM were extracted from the Traditional Chinese Medicine Systems Pharmacology Database and Analysis Platform, while the targets of CHD, anxiety, and depression were collected from the GeneCards database. The networks of active ingredients-disease targets were acquired according to the Cytoscape 3.6.1. The active ingredients that most related with CHD, anxiety, and depression simultaneously were acquired by matching ingredients-disease targets. Also, the main potential mechanisms of the primary active compounds (top 10) were summarized by the database of Web of Science.

## Results

### Literature Search Results

**FIGURE 1:**
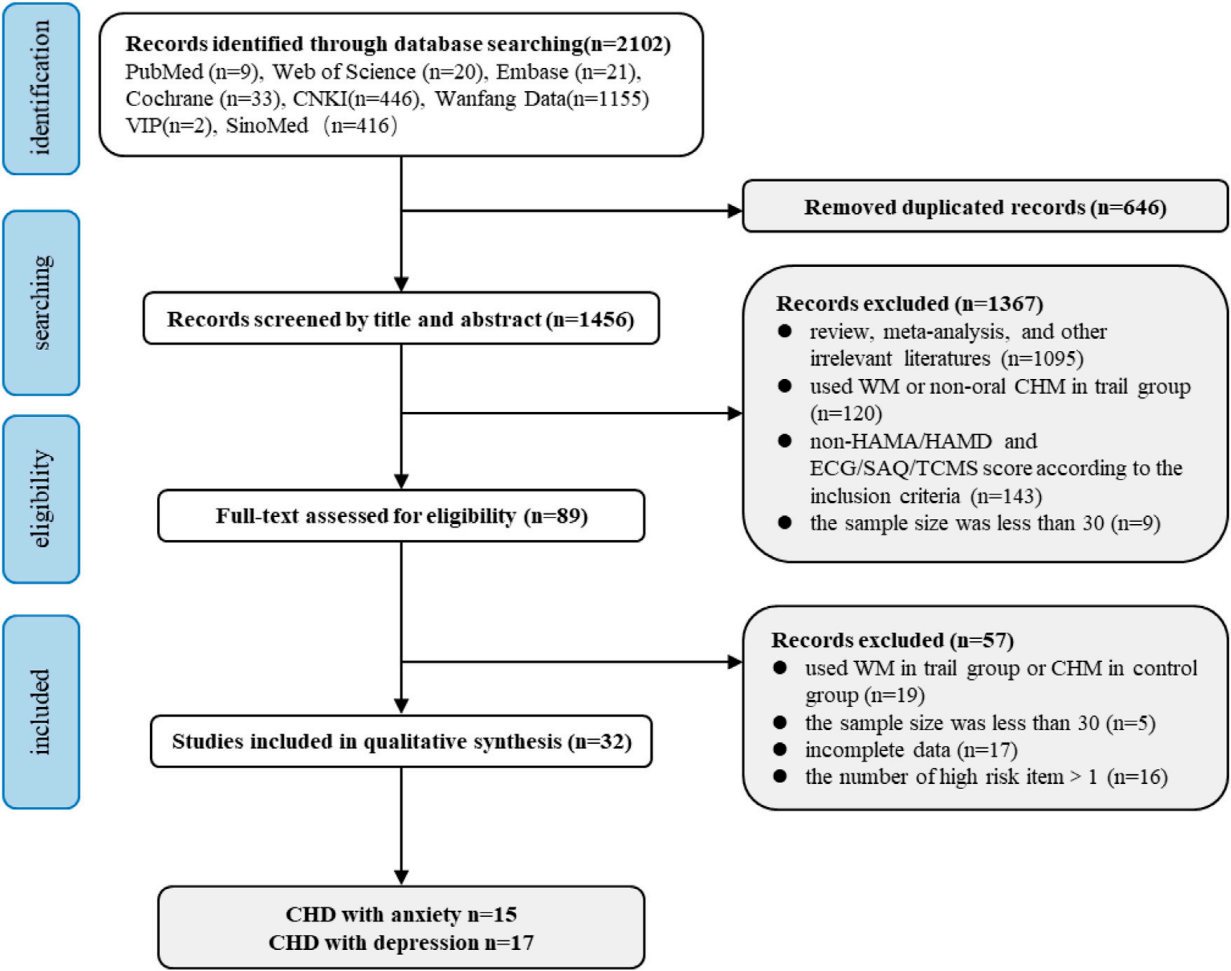
Flow diagram of literature search.

A total of 2,102 records were identified from eight electronic databases. Thirty-two studies met the inclusion criteria, and 2070 studies were excluded due to 1) irrelevant studies; 2) nonclinical studies; 3) review, meta-analysis, and conference abstracts; 4) sample size was less than 30; 5) using WM in trial groups; 6) non-HAMA or HAMD for evaluating the efficacy of anxiety or depression; 7) non-ECG or AS or AF or TCMS score for evaluating the efficacy of CHD; and 8) articles with incomplete data or more than one high-risk item. The specific screening process is illustrated in Figure 1.

### Study and Patient Characteristics

Thirty-two studies included 15 studies on CHD with anxiety (Mo et al., 2016; Li G. Y. et al., 2017; Guo, 2017; Qi and Song, 2017; Zhang et al., 2017; Qin, 2018; Wang C., 2018; Chen, 2019; Dong, 2019; Yang, 2019; Zhang, 2019; Zhao et al., 2019; Jin et al., 2021; Wang et al., 2021; Zhang and Jin, 2021) and 17 studies on CHD with depression (Sun, 2011; Lin, 2012; Zhang et al., 2012; Qin and Liu, 2013; Zhu, 2013; Gu et al., 2014; Shang et al., 2014; Mu, 2015; Shi et al., 2016; Li F. E. et al., 2017; Su, 2017; Shi, 2018; Wang Y. et al., 2018; Wang D. D., 2018; Lu, 2019; Huang et al., 2020; Zhang et al., 2020). All studies accounted for baseline comparability, and the patients’ overall characteristics are summarized in Tables 2A,B. Subjects of CHD who were diagnosed as stable angina (SA), unstable angina (UA), acute myocardial infarction (AMI), non-ST segment elevation myocardial infarction (NSTEMI), or post-percutaneous coronary intervention (post-PCI) were also evaluated by HAMA (Mo et al., 2016; Li G. Y. et al., 2017; Guo, 2017; Qi and Song, 2017; Zhang et al., 2017; Qin, 2018; Wang C., 2018; Chen, 2019; Dong, 2019; Yang, 2019; Zhang, 2019; Zhao et al., 2019; Jin et al., 2021; Wang et al., 2021; Zhang and Jin, 2021) and HAMD (Sun, 2011; Lin, 2012; Zhang et al., 2012; Qin and Liu, 2013; Zhu, 2013; Gu et al., 2014; Shang et al., 2014; Mu, 2015; Shi et al., 2016; Li F. E. et al., 2017; Su, 2017; Shi, 2018; Wang Y. et al., 2018; Wang D. D., 2018; Lu, 2019; Huang et al., 2020; Zhang et al., 2020). Secondary prevention drugs for CHD were used in all studies.

**TABLE 2A:** Research characteristics of the CHD with anxiety.

Study (year)	Disease		N (male/female), Mean age (years)		Basic treatment	Interventions		Duration of treatment	Outcome index	Intergroup difference
	CHD	Anxiety	Control group	Trial group		Control group	Trial group			
Mo 2016 Mo et al. (2016)	Post-PCI	HAMA≥14	32 (17/15) 60	33 (19/14) 58	Unspecified	N	Wuling capsule	8 weeks	1. HAMA score	1. <0.05
									2. ECG efficacy	2. <0.05
Guo 2017 Guo, (2017)	SA	14 ≤ HAMA<29	30 (8/22) 56.97 ± 7.51	30 (9/21) 58.00 ± 8.54	A1, A3, E	N	Shenchai Shuxin decoction	4 weeks	1. HAMA score&efficacy	1. <0.05 and <0.05
									2. ECG efficacy	2. >0.05
									3. AS score	3. <0.05
									4. AF score	4. <0.05
									5. TCMS score and efficacy^a^	5. <0.05 and <0.05
Li 2017 Li G. Y. et al. (2017)	Post-PCI	HAMA ≤ 29	34	33	A1, A2, B, C1	N	Tiaogan Jianpi Tongyang decoction	2 weeks	1. HAMA score	1. <0.01
									2. Efficacy of angina^a^	2. <0.01
Qi 2017 Qi and Song (2017)	Post-PCI	HAMA > 14	45 (21/24) 54 ± 10.6	45 (22/23) 51 ± 9.4	A1, C1	Flupentixol and melitracen tablets	Jieyu Tongmai granule	2 weeks	1. HAMA efficacy	1. >0.05
									2. ECG efficacy	2. <0.01
Zhang 2017 Zhang et al. (2017)	SA	HAMA ≥ 14	40 (19/21) 59.60 ± 3.93	40 (21/19) 60.08 ± 5.21	A1, A3, C1	N	Wuling capsule	12 weeks	1. HAMA efficacy	1. <0.05
									2. Efficacy of angina^a^	2. <0.05
Qin 2018 Qin (2018)	Post-PCI	HAMA>14	31 (22/9) 68.87 ± 8.60	31 (23/8) 70.74 ± 9.30	A1, B, C1, M	Flupentixol and melitracen tablets	Yuxin decoction	30 days	1. HAMA score	1. >0.05
									2. TCMS score^b^	2. <0.01
Wang 2018 Wang C. (2018)	SA	14 ≤ HAMA < 29	30 (13/17) 56.20 ± 12.47	30 (11/19) 58.27 ± 12.96	A1, A2, A3, B, C1, C2, M	N	Chaihu Longgu Muli granule	4 weeks	1. HAMA score	1. <0.05
									2. Efficacy of angina^a^	2. <0.05
Chen 2019 Chen (2019)	SA	14 < HAMA < 29	30 (16/14) 61.17 ± 6.06	30 (17/13) 61.43 ± 5.85	A1, A3, B, C1, E	N	Chaihu Jieyu decoction	4 weeks	1. HAMA score&efficacy	1. <0.05 and <0.05
									2. ECG efficacy	2. <0.05
									3. TCMS score and efficacy^a^	3. <0.05 and <0.05
Dong 2019 Dong (2019)	SA	14 < HAMA < 29	30 (13/17) 58.10 ± 7.32	30 (15/15) 58.47 ± 6.05	A1, A3, C1	N	Danqi Anshen decoction	4 weeks	1. HAMA score and efficacy	1. <0.01and<0.05
									2. ECG efficacy	2. >0.05
									3. AS score	3. <0.01
									4. AF score	4. <0.01
									5. TCMS score and efficacy^a^	5. <0.01and<0.05
Yang 2019 Yang (2019)	SA	14 ≤ HAMA < 29	32 (22/10) 62.33 ± 9.42	32 (19/13) 60.15 ± 8.54	A1, A2, A3, A4, B, C1, C2, M	N	Chaihu Longgu Muli granule	4 weeks	1. HAMA score and efficacy	1. <0.05 and <0.05
									2. TCMS score and efficacy	2. <0.05 and <0.05
Zhang 2019 Zhang (2019)	UA	14 ≤ HAMA < 21	41 59.76 ± 10.39	42 59.79 ± 9.54	A1, C1	N	Jiangqi Dayu decoction	3 weeks	1. HAMA score and efficacy	1. <0.01 and <0.05
									2. ECG efficacy	2. >0.05
									3. TCMS score and efficacy^a^	3. <0.01 and <0.05
Zhao 2019 Zhao et al. (2019)	Post-PCI	HAMA ≥ 7	40 (14/26) 75.23 ± 10.41	40 (18/22) 76.75 ± 12.52	A1, A2, A3, B, C1, C2, E	N	Xinling pill	3 months	1. HAMA score	1. <0.05
									2. ECG efficacy	2. <0.05
									3. AS score	3. <0.05
									4. AF score	4. <0.05
									5. TCMS efficacy^a^	5. <0.05
Jin 2021 Jin et al. (2021)	SA	14 < HAMA < 29	40 (19/21) 54.67 ± 3.28	40 (18/22) 55.24 ± 4.75	A1, A3, C1	Diazepam	Shuxin decoction	4 weeks	1. HAMA score and efficacy	1. <0.05 and <0.05
									2. AS score	2. <0.05
									3. AF score	3. <0.05
									4. TCMS score and efficacy^a^	4. <0.05
Wang 2021 Wang et al. (2021)	SA	HAMA ≥ 14	43 (31/12) 55.16 ± 11.49	43 (28/15) 59.11 ± 11.40	A1, A3, B, C1, D, M	N	Shuxin oral liquid	12 weeks	1. HAMA score	1. <0.05
									2. AS score	2. <0.05
									3. AF score	3. <0.05
									4. TCMS score^b^	4. <0.05
Zhang 2021 Zhang and Jin (2021)	SA	14 ≤ HAMA < 29	40 (21/19) 66.8 ± 5.6	42 (22/20) 67.5 ± 5.2	A1, A3, B, C1, E	Lorazepam	Xuefu Zhuyu decoction and Yueju pill	4 weeks	1. HAMA efficacy	1. <0.05
									2. ECG efficacy	2. <0.05
									3. AS score	3. <0.05
									4. AF score	4. <0.05
									5. TCMS score^a^	5. <0.05

Note: A1, antiplatelets; A2, ACEI/ARB; A3, nitrate esters drugs; A4, anticoagulants; B, *β*-blocker; C1, statins; C2, Ca antagonists; CHD, coronary heart disease; D, antidiabetic drugs; E, regulate emotion; M, improve the metabolism; N, without intervention; PCI, percutaneous coronary intervention; SA, stable angina; TCMS, traditional Chinese medicine syndrome; UA, unstable angina

a The evaluation criteria refer to the guiding principles for clinical research of Chinese medicine from China.

b The evaluation criteria refer to other acceptable evaluation methods.

**TABLE 2B:** Research characteristics of the CHD with depression.

Study (year)	Disease		N (male/female), Mean age (years)		Basic treatment	Interventions		Duration of treatment	Outcome index	Intergroup difference
	CHD	Depression	Control group	Trial group		Control group	Trial group			
Sun (2011)	SA, UA, AMI	18 ≤ HAMD ≤ 34	30 (12/18) 61.80 ± 6.33	30 (10/20) 60.80 ± 6.34	A1, A3, A4, C1, E	N	Jieyu Anshen decoction	4 weeks	1. HAMD-24 score and efficacy	1. <0.01 and <0.05
									2. TCMS score and efficacy^b^	2. <0.01 and <0.05
Lin (2012)	SA, UA, NSTEMI, AMI	HAMD≥17	30 (16/14) 61.83 ± 7.95	30 (15/15) 61.77 ± 8.00	Unspecified	Placebo	Xiaoyao pill	1 month	1. HAMD-17 score	1. <0.05
									2. AS score	2. >0.05
									3. AF score	3. >0.05
Zhang et al. (2012)	SA	HAMD	35 (11/24) 68.67 ± 9.89	36 (12/24) 68.63 ± 8.41	A3	Fluoxetine hydrochloride	Jiawei Shengdan Louxie Sini granule	4 weeks	1. HAMD score	1. <0.01
									2. ECG efficacy	2. <0.01
									3. TCMS efficacy^a^	3. <0.01
Qin and Liu (2013)	SA	HAMD-24 > 7	30	31	A3	Fluoxetine hydrochloride	Tongxin Jieyu granule	8 weeks	1. HAMD-24 score	1. <0.05
									2. ECG efficacy	2. <0.01
									3. TCMS score and efficacy^a^	3. <0.05 and <0.01
									4. Efficacy of angina^a^	4. <0.05
Zhu 2013 Zhu (2013)	UA	HAMD	30 (16/14) 61.4 ± 8.2	30 (18/12) 62.8 ± 9.5	A1, A3, A4. B, C1	Flupentixol and melitracen tablets	Jieyu granule	4 weeks	1. HAMD-17 score	1. >0.05
									2. ECG efficacy	2. >0.05
									3. TCMS score and efficacy^a^	3. >0.05 and >0.05
									4. Efficacy of angina^a^	4. >0.05
Gu 2014 Gu et al. (2014)	SA, UA	HAMD	30 (18/12) 64.12 ± 7.33	30 (19/11) 63.32 ± 8.16	A1, A3, B, C1	N	Shugan Jieyu decoction	4 weeks	1. HAMD efficacy	1. <0.05
									2. ECG efficacy	2. <0.05
									3. Efficacy of angina^a^	3. <0.05
Shang 2014 Shang et al. (2014)	Post-PCI	HAMD-17 > 20	30 (12/18) 58.0	30 (14/16) 56.5	Unspecified	Fluoxetine hydrochloride	Chaihu Longgu Muli decoction	4 weeks	1. HAMD-17 score	1. <0.05
									2. TCMS efficacy^a^	2. <0.05
Mu 2015 Mu, (2015)	Post-PCI	18 ≤ HAMD≤24	30 (22/8) 70.9 ± 12.3	30 (23/7) 72.8 ± 11.4	A1, A3, A4, C1, E	N	Yangxin Jieyu decoction	4 weeks	1. HAMD-24 score and efficacy	1. <0.01 and <0.01
									2. ECG efficacy	2. <0.05
									3. TCMS score and efficacy^a^	3. <0.01 and <0.01
Shi 2016 Shi et al. (2016)	Post-PCI	8<HAMD-24 < 34	31	32	A1	Escitalopram	Jieyu Yongmai recipe	8 weeks	1. HAMD-24 score	1. >0.05
									2. AS score	2. <0.05
									3. AF score	3. <0.05
									4. TCMS score^a^	4. <0.05
Li 2017 Li F. E. et al. (2017)	Post-PCI	HAMD≥20	40 (17/23) 64.1	40 (21/19) 62.3	Unspecified	Flupentixol and melitracen tablets	Jiawei Wendan decoction	12 weeks	1. HAMD-17 score	1. <0.05
									2. TCMS efficacy^a^	2. >0.05
Su 2017 Su (2017)	Post-PCI, post-CABG	HAMD-24	30 (15/15) 73.17 ± 9.84	30 (16/14) 70.77 ± 8.53	A1, A3, C1, E	N	Suanzaoren decoction	8 weeks	1. HAMD-24 score and efficacy	1. <0.01 and <0.01
									2. AS score	2. <0.01
									3. AF score	3. <0.01
									4. TCMS score and efficacy^a^	4. <0.01 and <0.05
Wang1 2018 Wang D. D. (2018)	SA	20<HAMD-24 ≤ 35	34 (18/16) 64.36 ± 7.20	36 (16/20) 61.44 ± 9.45	A1, A3, B, C1	Flupentixol and melitracen tablets	Dachaihu decoction	4 weeks	1. HAMD-24 score&efficacy	1. <0.05 and <0.05
									2. AS score	2. <0.05
									3. AF score	3. <0.05
									4. TCMS score and efficacy^a^	4. <0.05 and <0.05
Wang2 2018 Wang Y. et al. (2018)	SA, UA	HAMD≥20	140 (64/76) 56.3 ± 8.8	140 (61/79) 55.2 ± 9.1	A1, A2, A3, B, C1, C2	Escitalopram	Buxinqi decoction	8 weeks	1. HAMD score	1. <0.01
									2. Episodes of angina	2. <0.01
									3. Duration of angina	3. <0.01
Shi 2018 Shi (2018)	UA	HAMD-17	34 (14/20) 62.77 ± 8.77	34 (13/21) 61.59 ± 7.79	A1, A3, B, C1	N	Tongmai Sanyu granule	2 weeks	1. HAMD-17 score	1. <0.05
									2. ECG efficacy	2. <0.05
									3. TCMS score and efficacy^a^	3. <0.05 and <0.05
Lu 2019 Lu (2019)	Post-PCI	HAMD	41 (27/14) 63.12 ± 7.55	38 (21/17) 64.13 ± 6.72	A1, C1	Flupentixol and melitracen tablets	Jieyu Shugan Tongmai recipe	2 weeks	1. HAMD-24 score&efficacy	1. >0.05 and >0.05
									2. TCMS score^a^	2. <0.05
Huang 2020 Huang et al. (2020)	IHD	HAMD	50 (32/18) 67.38 ± 8.41	50 (36/14) 68.68 ± 7.27	A1, A3, B, C1, E	N	Guanxinning tablet	1 month	1. HAMD-17 score and efficacy	1. <0.05 and <0.05
									2. ECG efficacy	2. <0.05
Zhang 2020 Zhang et al. (2020)	SA	8<HAMD-17 < 24	30 (17/13) 62.73 ± 8.57	30 (15/15) 60.77 ± 7.55	A1, A3, B, C1	N	Huatan Quyu recipe	4 weeks	1. HAMD-17 score	1. <0.05
									2. ECG efficacy	2. >0.05
									3. TCMS score^b^	3. <0.05

Note: A1, antiplatelets; A2, ACEI/ARB; A3, nitrate esters drugs; A4, anticoagulants; AMI, acute myocardial infarction; B, *β*-blocker; C1, statins; C2, Ca antagonists; CABG, coronary artery bypass grafting; CHD, coronary heart disease; E, regulate emotion; IHD, ischemic heart disease; N, without intervention; NSTEMI, non-ST, segment elevation myocardial infarction; PCI, percutaneous coronary intervention; SA, stable angina; TCMS, traditional Chinese medicine syndrome; UA, unstable angina

a The evaluation criteria refer to the guiding principles for clinical research of Chinese medicine from China.

b The evaluation criteria refer to other acceptable evaluation methods.

For control groups of CHD with anxiety, four studies used flupentixol and melitracen tablets (Qi and Song, 2017; Qin, 2018), diazepam (Jin et al., 2021), and lorazepam (Zhang and Jin, 2021), while nine studies used fluoxetine hydrochloride (Zhang et al., 2012; Qin and Liu, 2013; Shang et al., 2014), flupentixol and melitracen tablets (Zhu, 2013; Li F. E. et al., 2017; Wang D. D., 2018; Lu, 2019), and escitalopram (Shi et al., 2016; Wang Y. et al., 2018) in the CHD with depression. No WM were used in control groups in the remaining researches except for the study by Lin et al. who used a placebo (Lin, 2012). CHM was used in trial groups and the details are shown in Table 1. The treatment course in all studies varied from 2 weeks to 3 months. The primary efficacy endpoints, including the score and efficacy of HAMA and HAMD, ECG efficacy, AS score, and AF score, were extracted for this meta-analysis and systematic review. The score and efficacy of TCMS were also extracted for the evaluation as the secondary efficacy endpoint.

### Quality Assessment of Included Studies

The study methodological quality is concluded in Supplementary Table S1. Random allocation was used in all included studies. Five studies performed blind method (Lin, 2012; Mo et al., 2016; Li G. Y. et al., 2017; Wang C., 2018; Su, 2017), and blinded outcome assessment was conducted in two studies (Qi and Song, 2017; Zhang and Jin, 2021). Additionally, allocation concealment was used in three studies (Sun, 2011; Mo et al., 2016; Wang C., 2018).

### Efficacy of Chinese Herbal Medicine in Coronary Heart Disease With Anxiety

As shown in Table 2A, the score and efficacy of HAMA, ECG, AS, AF, and TCMS in trial groups in most studies possessed a significant improvement. However, there were also some different results. Two studies showed that there was no significant difference in the score or efficacy of HAMA between trial groups and flupentixol and melitracen tablet-treated groups (Qi and Song, 2017; Qin, 2018). Three studies reported that the efficacy of ECG in trial groups was not significantly different compared with blank control groups (Mo et al., 2016; Chen, 2019; Zhang, 2019). Thus, the primary endpoint results were pooled to further confirm the efficacy of CHM.

#### Efficacy of Chinese Herbal Medicine in Anxiety

**FIGURE 2:**
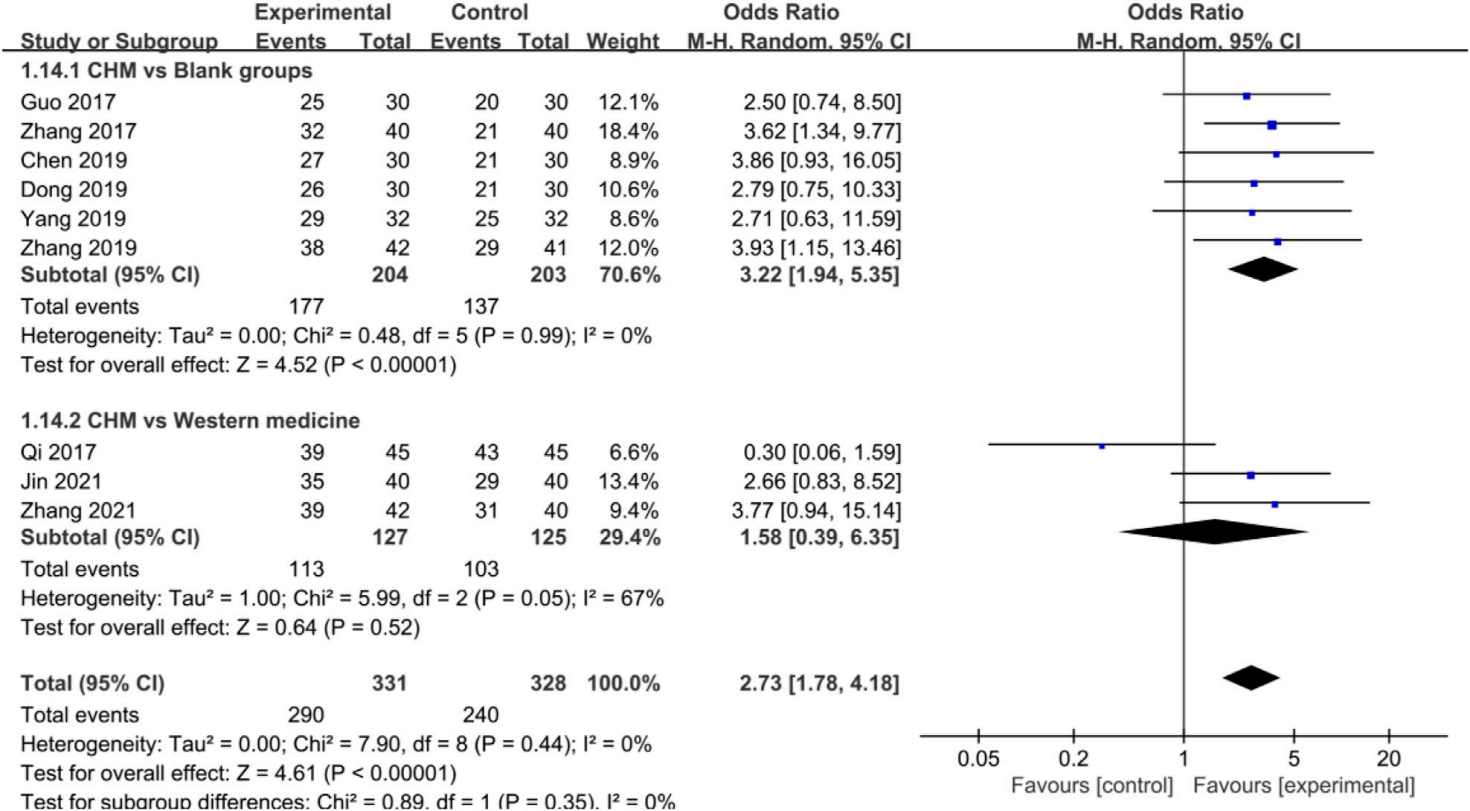
Forest plot: CHM improved HAMA efficacy in CHD with anxiety compared with control groups.

In Supplementary Figure S1, the HAMA score displayed significant heterogeneity due to scoring bias in different studies. Therefore, the efficacy of CHM for treating anxiety was further analyzed. Meta-analysis of nine studies showed a significant efficiency of CHM for improving anxiety [OR = 2.73, 95%CI (1.78, 4.18), *p* < 0.00001, I^2^ = 0%] (Figure 2), and the subgroup analysis based on whether the control group used WM or not was also performed. As shown in Figure 2, the results of subgroup analysis showed a favor for CHM in curing anxiety in CHD patients compared with blank control groups [OR = 3.22, 95%CI (1.94, 5.35), *p* < 0.00001, I^2^ = 0%], whereas the efficacy of CHM in treating anxiety was not inferior to that of WM [OR = 1.58, 95%CI (0.39, 6.35), *p* = 0.52, I^2^ = 67%]. Moreover, a repetitive meta-analysis by consecutively excluding each study in WM groups was performed. The study by Qi et al. was the main source of heterogeneous, but it was not removed because of reasonable research design.

#### Efficacy of Chinese Herbal Medicine in Coronary Heart Disease

**FIGURE 3:**
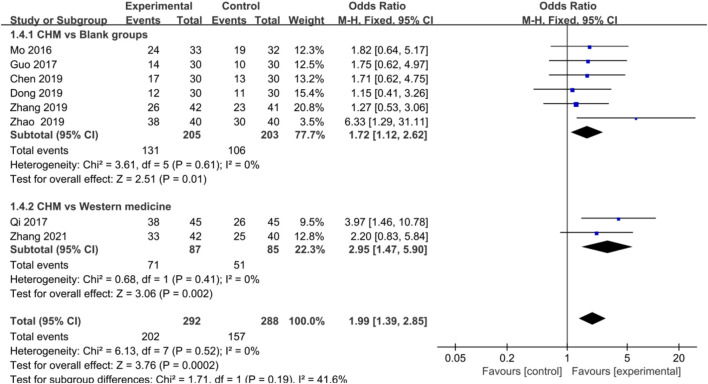
Forest plot: CHM improved ECG in CHD with anxiety compared with control groups.

Meta-analysis of eight studies showed that the improvement of ECG in CHD patients was significantly associated with CHM treatment [OR = 1.99, 95%CI (1.39, 2.85), *p* = 0.0002, I^2^ = 0%] (Figure 3). In addition, subgroup analysis showed a consistent result favoring CHM in improving CHD compared with blank [OR = 1.72, 95%CI (1.12, 2.62), *p* = 0.01, I^2^ = 0%] and WM groups [OR = 2.95, 95%CI (1.47, 5.90), *p* = 0.002, I^2^ = 0%] (Figure 3).

**FIGURE 4:**
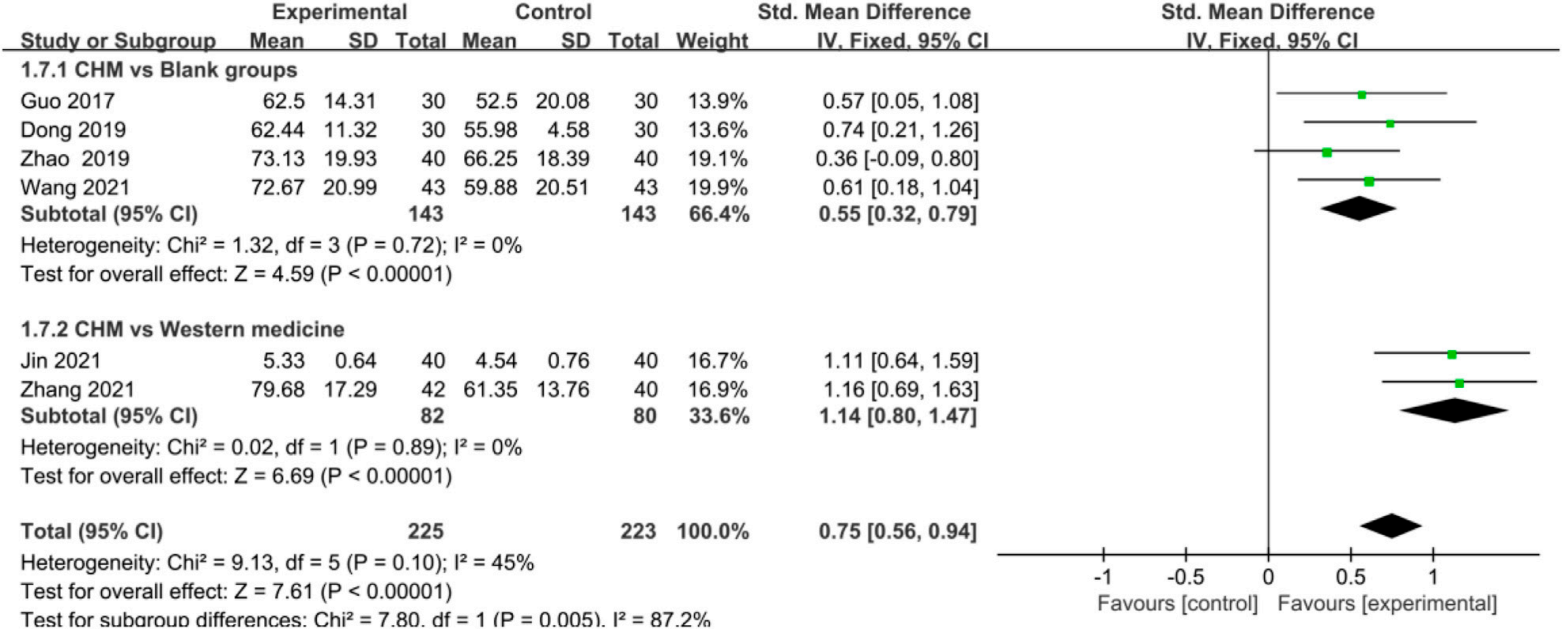
Forest plot: CHM for improved AS of CHD with anxiety compared with control groups.

**FIGURE 5:**
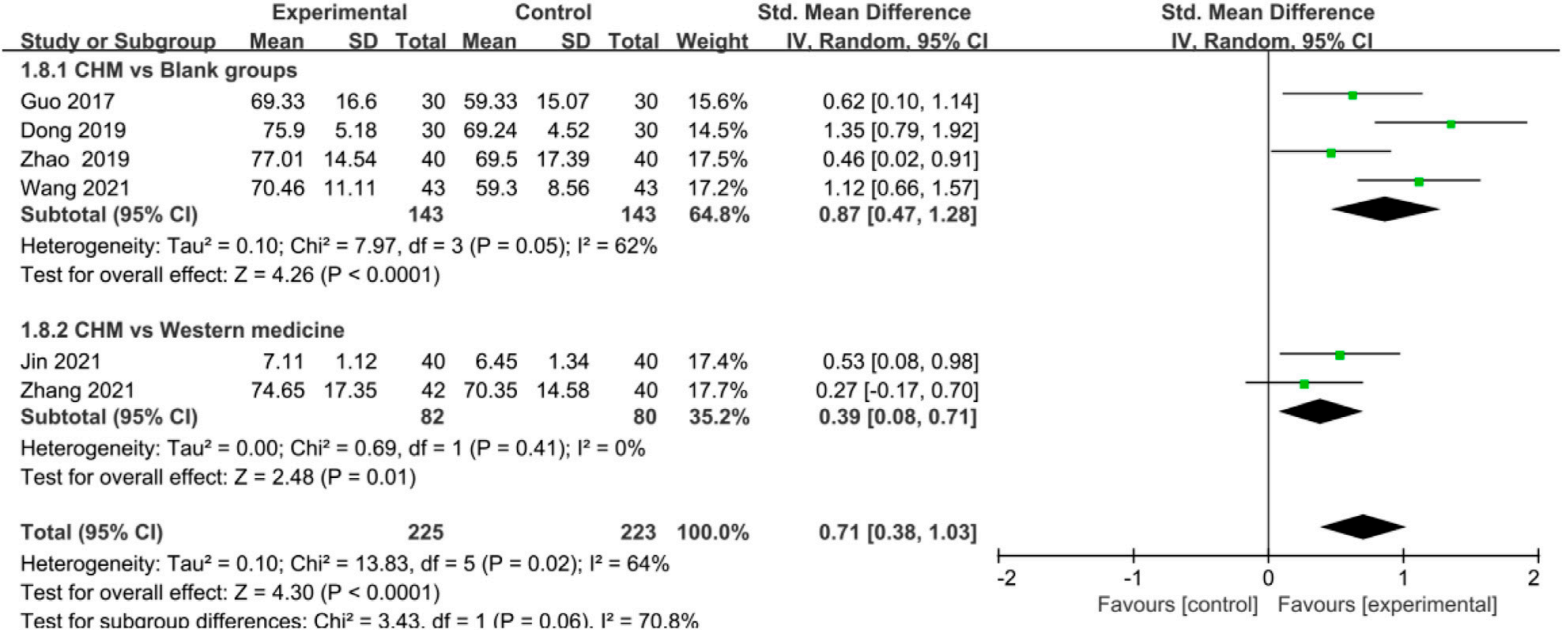
Forest plot: CHM for improved AF in CHD with anxiety compared with control groups.

In terms of improving AS and AF, CHM also showed a significant advantage in trial groups compared with control groups [AS: SMD = 0.75, 95%CI (0.56, 0.94), *p* < 0.00001, I^2^ = 45%; AF: SMD = 0.71, 95%CI (0.38, 1.03), *p* < 0.0001, I^2^ = 64%] (Figures 4, 5), blank groups [AS: SMD = 0.55, 95%CI (0.32, 0.79), *p* < 0.00001, I^2^ = 0%; AF: SMD = 0.87, 95%CI (0.47, 1.28), *p* < 0.0001, I^2^ = 62%], and WM groups [AS: SMD = 1.14, 95%CI (0.80, 1.47), *p* < 0.00001, I^2^ = 0%; AF: SMD = 0.39, 95%CI (0.08, 0.71), *p* = 0.01, I^2^ = 0%] (Figures 4, 5).

### Efficacy of Traditional Chinese Medicine in Coronary Heart Disease With Depression

As shown in Table 2B, most included studies showed a significant improvement in the score and efficacy of HAMD, ECG, AS, AF, and TCMS in treatment groups. However, some studies showed different results. Three studies reported that the score or efficacy of HAMD in CHM groups was no statistical difference between treatment and control groups using antidepressants (Zhu, 2013; Shi et al., 2016; Lu, 2019). Similarly, for the score or efficacy of ECG, angina, and TCMS, there were also no statistical differences between treatment and control groups (Zhu, 2013; Gu et al., 2014; Shang et al., 2014; Li F. E. et al., 2017; Zhang et al., 2020). Additionally, the study by Lin et al. was the only study that used placebo (Lin, 2012). The scores of AS and AF were not significantly different between the CHM and placebo group, but the result of the 36-item short form survey showed a superior benefit of CHM compared with placebo. In the study by Wang Y. et al. (2018), antidepressants and CHM both possessed obvious efficacy for treating CHD with depression, and antidepressants exhibited even more efficiency. Therefore, the primary endpoint results were pooled to further confirm the efficacy of CHM.

#### Efficacy of Chinese Herbal Medicine in Depression

**FIGURE 6:**
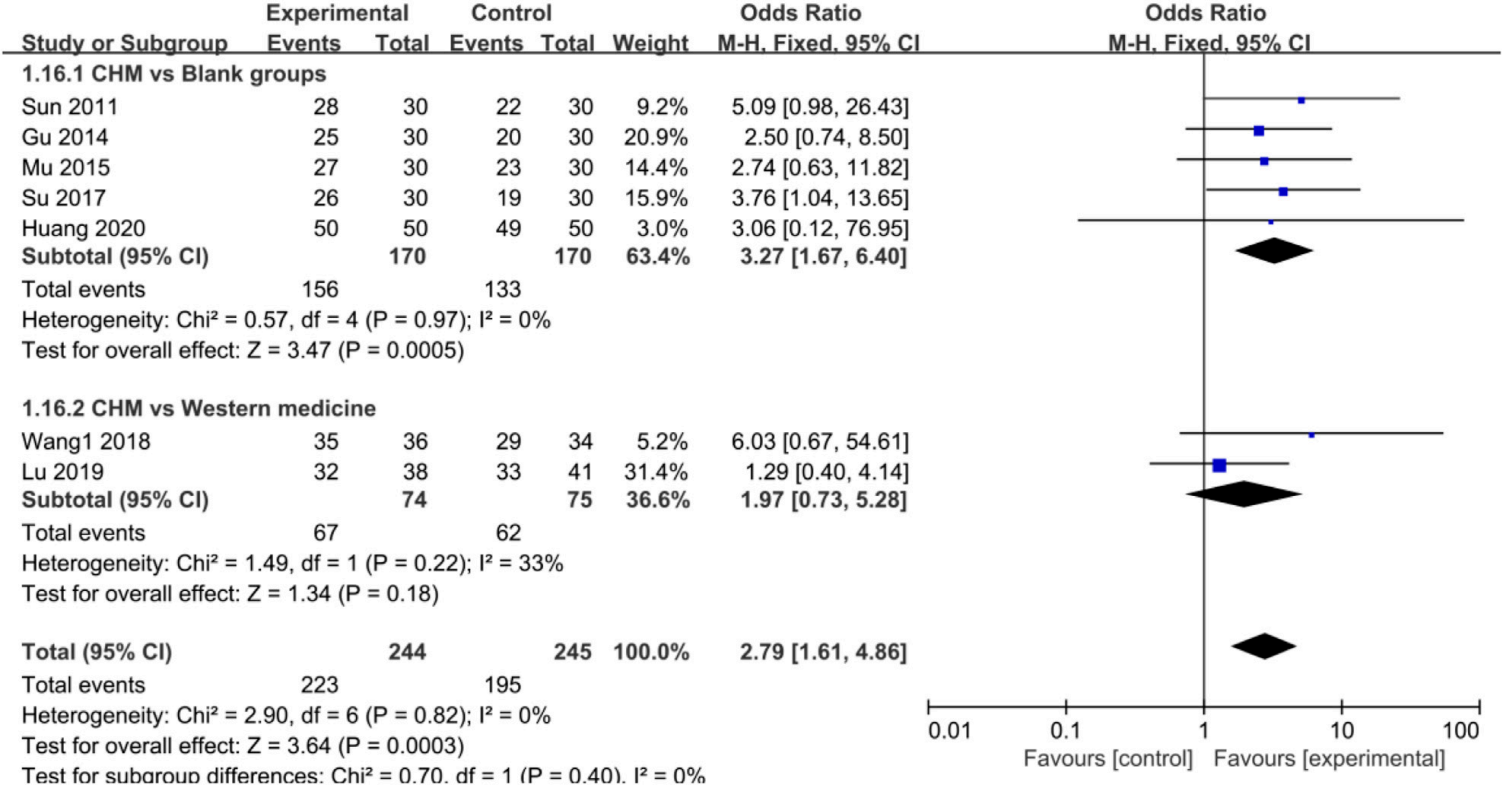
Forest plot: CHM improved HAMD efficacy in CHD with depression compared with control groups.

Meta-analysis of seven studies showed that CHM had a significant effect on treating depression compared with control groups [OR = 2.79, 95%CI (1.61, 4.86), *p* = 0.0003, I^2^ = 0%] (Figure 6). The results of subgroup analysis also revealed that the antidepressive effect was improved significantly compared with blank control groups [OR = 3.27, 95%CI (1.67, 6.40), *p* = 0.0005, I^2^ = 0%] but was the same as WM groups [OR = 1.97, 95%CI (0.73, 5.28), *p* = 0.18, I^2^ = 33%] (Figure 6).

#### Efficacy of Chinese Herbal Medicine in Coronary Heart Disease

**FIGURE 7:**
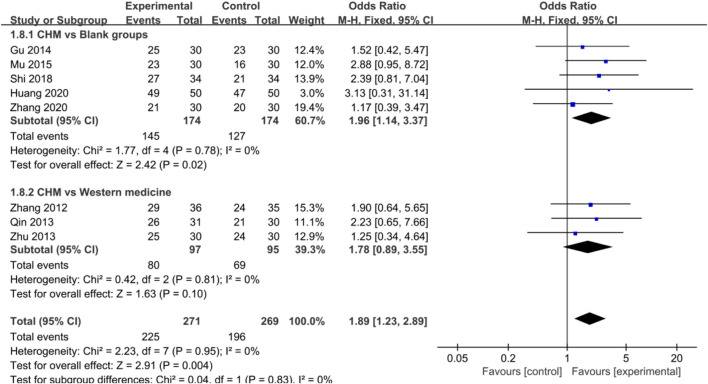
Forest plot: CHM improved ECG in CHD with depression compared with control groups.

Eight studies reported that CHM significantly improved ECG in CHD patients [OR = 1.89, 95%CI (1.23, 2.89), *p* = 0.004, I^2^ = 0%] (Figure 7). In addition, subgroup analysis showed a similar result favoring CHM in improving CHD compared with blank groups [OR = 1.96, 95%CI (1.14, 3.37), *p* = 0.02, I^2^ = 0%], but no statistical difference was found when comparing CHM with WM groups [OR = 1.78, 95%CI (0.89, 3.55), *p* = 0.10, I^2^ = 0%] (Figure 7).

**FIGURE 8:**
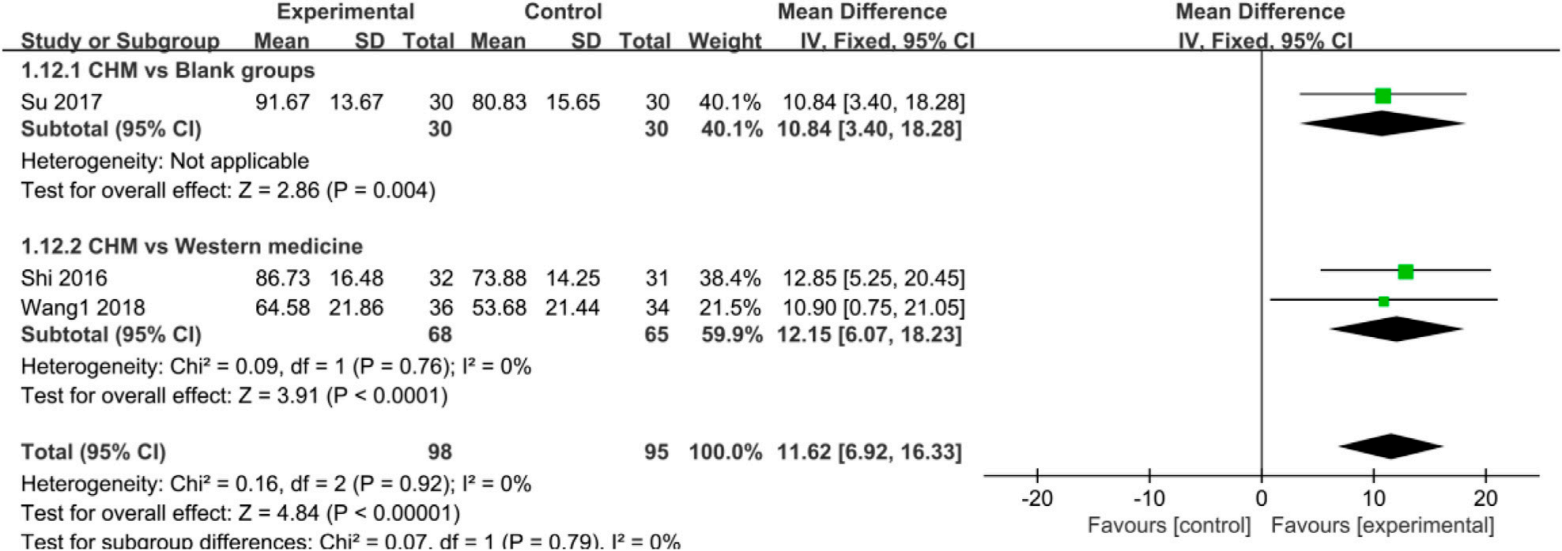
Forest plot: CHM for improved AS in CHD with depression compared with control groups.

**FIGURE 9:**
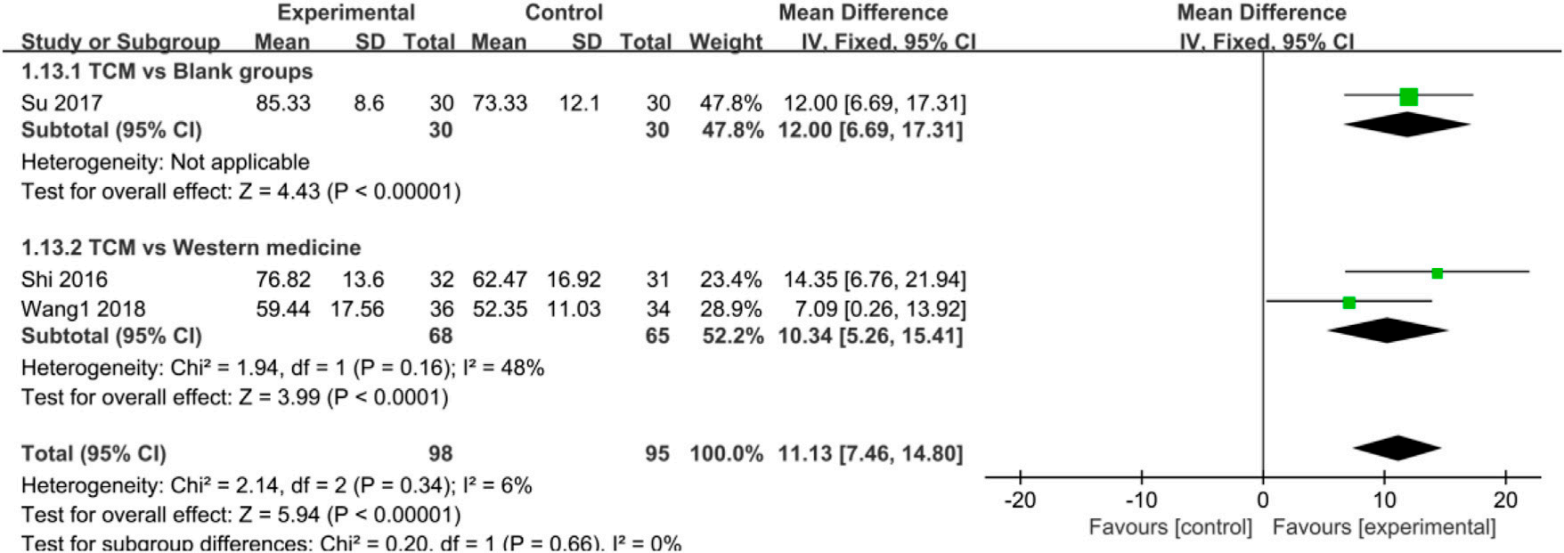
Forest plot: CHM improved AF in CHD with depression compared with control groups.

Regarding the efficacy of CHM in AS and AF, CHM also provided a more significant advantage compared with control groups [AS: SMD = 11.62, 95%CI (6.92, 16.33), *p* < 0.00001, I^2^ = 0%; AF: SMD = 11.13, 95%CI (7.46, 14.80), *p* < 0.00001, I^2^ = 6%] (Figures 8, 9), blank groups [AS: *p* = 0.004; AF: *p* < 0.00001], and WM [AS: SMD = 12.15, 95%CI (6.07, 18.23), *p* < 0.0001, I^2^ = 0%; AF: SMD = 10.34, 95%CI (5.26, 15.41), *p* < 0.0001, I^2^ = 48%] (Figures 8, 9).

### Comparisons of the Characteristics of Chinese Herbal Medicine in Coronary Heart Disease With Anxiety or Depression

Due to the promising results of CHM treatment observed in most included studies, the frequency statistics of CHM was analyzed to identify the commonly used drugs among different groups. The results showed that *Bupleurum chinense* DC. [Apiaceae; Bupleuri radix], *Glycyrrhiza uralensis* Fisch. ex DC. [Fabaceae; Glycyrrhizae radix et rhizoma], *Ligusticum chuanxiong* Hort. [Apiaceae; Chuanxiong rhizoma], *Salvia miltiorrhiza* Bunge [Lamiaceae; Salviae miltiorrhizae radix et rhizoma], *Angelica sinensis* (Oliv.) Diels [Apiaceae; Angelicae sinensis radix], *Paeonia lactiflora* Pall. [Paeoniaceae; Paeoniae radix alba], *Pinellia ternata* (Thunb.) Makino [Araceae; Pinelliae rhizoma], *Curcuma aromatica* Salisb. [Zingiberaceae; Curcumae radix], and *Citrus×aurantium* L. [Rutaceae; Aurantii fructus] were commonly used for treating CHD with anxiety or depression (Supplementary Table S2). Also, the CHM with a frequency not less than three were selected for the networks of active ingredients-disease targets, and the results demonstrated the efficacy of CHM for CHD with anxiety or depression (Supplementary Figure S2). Furthermore, the primary active ingredients of these CHM that could act on the targets of CHD, anxiety, and depression simultaneously were analyzed by matching ingredients disease targets. The results showed the active ingredients including quercetin, kaempferol, luteolin, beta-sitosterol, puerarin, stigmasterol, isorhamnetin, baicalein, tanshinone IIa, and nobiletin were most closely related to the targets of CHD, anxiety, and depression based on degree centrality, and the top 10 ingredients are shown in Table 3. These active compounds could either act on the targets of CHD, anxiety, and depression simultaneously or be extracted from varieties CHM.

**TABLE 3:** Mechanisms of main active components of CHM on CHD with anxiety or depression.

Active ingredient	Source	Structure	Models	Related mechanisms	References
Quercetin	*Bupleurum chinense* DC. [Apiaceae; Bupleuri radix], *Cyperus rotundus* L. [Cyperaceae; Cyperi rhizoma], *Ziziphus jujuba* Mill. [Rhamnaceae; Jujubae fructus], *Corydalis yanhusuo* (Y.H.Chou & Chun C.Hsu) W.T.Wang ex Z.Y.Su and C.Y.Wu [Papaveraceae; Corydalis rhizoma], *Carthamus tinctorius* L. [Asteraceae; Carthami flos], *Gardenia jasminoides* J.Ellis [Rubiaceae; Gardeniae fructus], *Glycyrrhiza uralensis* Fisch. ex DC. [Fabaceae; Glycyrrhizae radix et rhizoma], *Astragalus mongholicus* Bunge [Fabaceae; Astragali radix]	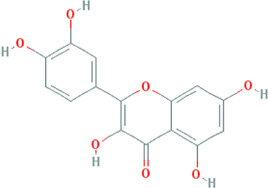	**CHD:** MI rat TNF-*α*-HUVEC	1. Anti damage/apoptosis (LDH, CK-MB, cTnI, Bax, cleaved caspase-3⍗)	Albadrani et al. (2020), Chen T. et al. (2020), Gma et al. (2021)
				2. Anti-inflammation (TNF-*α*, IL-6, VCAM-1, ICAM-1, E-selectin, AP-1⍗)	
				3. Antioxidative stress (MDA, ROS, mtPTP, cytochrome-C⍗; Nrf2, GSH, SOD, MnSOD⍐)	
				4. Antifibrosis (*α*-SMA, AngII, COL1A1, COL3A1⍗; Smad7, BMP7⍐)	
				5. Signal pathways (TGF-*β*1/Smad3, NF-kB, JAK-STAT3)	
			**Anxiety:** LPS-anxiety rat mTBI mouse SIA mouse	1. Anti-inflammation (IL-1*β*/6, cyclooxygenase-2, iNOS⍗)	Samad et al. (2018), Kosari-Nasab et al. (2019), Lee et al. (2020)
				2. Antioxidative stress (MDA⍗; CAT, GSH-Px, SOD⍐)	
				3. Maintaining neurotransmitters homeostasis (ACTH, Cort⍗; 5-HT, BDNF, ACh⍐)	
				4. Signal pathways (NF-κB)	
			**Depression:** CUMS mouse/rat	1. Anti-inflammation (IL-1*β*, TNF-*α*, iNOS⍗)	Guan Y. et al. (2021), Guan T. et al. (2021), Ma et al. (2021)
				2. Antioxidative stress (MAO, MDA⍗; GSH, GSHPx, CAT, SOD, GST, Nrf-2⍐)	
				3. Maintaining neurotransmitters homeostasis (BDNF⍐)	
				4. Signal pathways (FoxG1/CREB/BDNF, PI3K/AKT/HO-1)	
Kaempferol	*Bupleurum chinense* DC. [Apiaceae; Bupleuri radix], Paeonia lactiflora Pall. [Paeoniaceae; Paeoniae radix alba], Cyperus rotundus L. [Cyperaceae; Cyperi rhizoma], Gardenia jasminoides J.Ellis [Rubiaceae; Gardeniae fructus], *Glycyrrhiza uralensis* Fisch. ex DC. [Fabaceae; Glycyrrhizae radix et rhizoma], *Astragalus mongholicus* Bunge [Fabaceae; Astragali radix], *Carthamus tinctorius* L. [Asteraceae; Carthami flos]	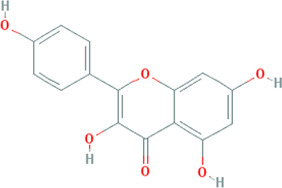	**CHD:** I/R DM rat ox-LDL-HUVECs	1. Anti-damage/apoptosis (Bax, cleaved-caspase-3, TUNEL, p38⍗; Bcl-2⍐)	Suchal et al. (2017), Li et al. (2021)
				2. Anti-inflammation (IL-1*β*/6, TNF-*α*⍗)	
				3. Antioxidative stress (ROS, MDA⍗; SOD⍐)	
				4. Signal pathways (circNOL12/miR-6873–3p/FRS2, NF-kB, AGE-RAGE/MAPK)	
			**Anxiety**: CS_1_+US rat	1. Regulating endocannabinoid system (FAAH enzyme⍗)	Ahmad et al. (2020)
			**Depression**: CSDS mouse	1. Anti-inflammation (IL-1*β*, TNF-*α*, CD11b⍗)	Gao W. et al. (2019)
				2. Antioxidative stress (MDA⍗; SOD, CAT, GST, GSH-Px⍐)	
				3. Signal pathways (AKT/*β*-catenin cascade)	
Luteolin	*Codonopsis pilosula* (Franch.) Nannf. [Campanulaceae; Codonopsis radix], *Cyperus rotundus* L. [Cyperaceae; Cyperi rhizoma], *Salvia miltiorrhiza *Bunge [Lamiaceae; Salviae miltiorrhizae radix et rhizoma], *Platycodon grandiflorus *(Jacq.) A.DC. [Campanulaceae; Platycodonis radix], *Carthamus tinctorius* L. [Asteraceae; Carthami flos]	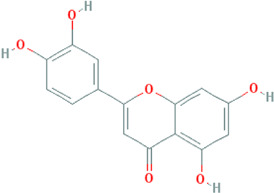	**CHD**: I/R-rats/mouse H_2_O_2_-H9C2 H/R-H9C2	1. Anti-damage/apoptosis (LDH, CK-MB,cTnI, Bax, caspase-1/3/9, cleaved-caspase-3, TUNEL⍗; Bcl-2⍐)	Yu et al. (2015), Hu et al. (2016), Wei et al. (2018), Hu et al. (2020), Zhao et al. (2020)
				2. Anti-inflammation (IL-1*β*/6, TNF-*α*, ASC⍗)	
				3. Antioxidative stress (ROS, MDA, MPO, p47-phox⍗; SOD, GSH, PRX II, Mn-SOD⍐)	
				4. Regulating autophagy (Mst1, p-Mst1, P62⍗; LC3-II, Beclin-1⍐)	
				5. Improving mitochondria function (ATP, CS_3_, complexes I/II/III/IV/V activities⍐)	
				6. Signal pathways (Sirt1/NLRP3/NF-κB; Sp1/SERCA2a, p38MAPK, JNK, ERK1/2)	
			**Anxiety**: male Swiss mouse	1. Maintaining neurotransmitters homeostasis (luteolin’s metabolites might show a higher affinity for the BDZ-R, and the anxiolytic-like effects through a GABAergic mechanism)	Coleta et al. (2008)
			**Depression**: OID rat + HNC male ICR mouse	1. Maintaining neurotransmitters homeostasis (BDNF, 5-HT⍐; PMAT⍗, GABAA receptor-Cl ion channel complex⍐)	de la Peña et al. (2014), Zhu et al. (2019)
Beta-sitosterol	*Citrus×aurantium* L. [Rutaceae; Aurantii fructus], *Angelica sinensis* (Oliv.) Diels [Apiaceae; Angelicae sinensis radix], *Paeonia lactiflora* Pall. [Paeoniaceae; Paeoniae radix alba], *Pinellia ternata* (Thunb.) Makino [Araceae; Pinelliae rhizoma], *Neolitsea cassia* (L.) Kosterm. [Lauraceae; Cinnamomi ramulus], *Cyperus rotundus* L. [Cyperaceae; Cyperi rhizoma], *Ziziphus jujuba* Mill. [Rhamnaceae; Jujubae fructus], *Zingiber officinale *Roscoe [Zingiberaceae; Zingiberis rhizoma recens], *Curcuma longa* L. [Zingiberaceae; Curcumae longae rhizoma], *Gardenia jasminoides* J.Ellis [Rubiaceae; Gardeniae fructus], *Wurfbainia villosa* (Lour.) Skornick. and A.D.Poulsen [Zingiberaceae; Amomi fructus], *Paeonia anomala subsp. veitchii* (Lynch) D.Y.Hong and K.Y.Pan [Paeoniaceae; Paeoniae radix rubra], *Carthamus tinctorius* L. [Asteraceae; Carthami flos], *Scutellaria baicalensis *Georgi [Lamiaceae; Scutellariae radix]	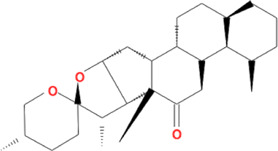	**CHD**: I/R SD rat H/R H9C2	1. Anti-damage/apoptosis (caspase-3/9⍗; Bcl-2⍐)	Lin et al. (2020)
				2. Antioxidative stress (ROS⍗)	
				3. Signal pathways (NF-κB; PPAR*γ*)	
			**Anxiety**: male Swiss mouse	1. Regulating of nervous system (anxiolytic-like action in 1–10 mg/kg and a sedative response in 30 mg/kg)	Aguirre-Hernández et al. (2007)
			**Depression**: Adult male ICR mouse	1. Maintaining neurotransmitters homeostasis (5-HT, 5-HIAA, NE, DA, GABAergic⍐)	Zhao et al. (2016), Yin et al. (2018)
Puerarin	*Bupleurum chinense* DC. [Apiaceae; Bupleuri radix]	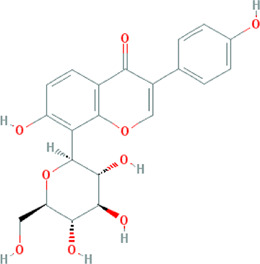	**CHD:** CHD rat I/R mouse	1. Anti-damage/apoptosis (CK, CK-MB, LDH, cTnT, cleaver caspase-1/3, Bax⍗; Bcl-2⍐)	Wang Z. K. et al. (2020), Zhao L. et al. (2021)
				2. Anti-inflammation (Lp-PLA2, TNF-*α*, CRP, IL-1*β*/6/18, NLRP3⍗; FXR⍐)	
				3. Antioxidative stress (MDA⍗; SOD⍐)	
				4. Reducing lipid (TC, DL, TG, ox-LDL⍗; HDL⍐)	
				5. Signal pathways (AKT/STAT3, SIRT1/NF-κB)	
			**Anxiety:** Male rat I/R rat	1. Anti-damage/apoptosis (cleaved-caspase-3⍗)	Tao et al. (2017), Qiu et al. (2018)
				2. Maintaining neurotransmitters homeostasis (allopregnanolone⍐; serotonin (5-HT) ⍐)	
				3. Signal pathways (PI3K/Akt1/GSK-3*β*/MCL-1)	
			**Depression:** CS_2_ mouse Male C57BL/6J mouse CUS rat	1. Anti-inflammation (COX-2, IL-1*β*/6, TNF-*α*⍗)	Qiu et al. (2017), Huang et al. (2018), Cheng et al. (2019)
				2. Maintaining neurotransmitters homeostasis (Cort, CRH, ACTH⍗; 5-HT, 5-HIAA, BDNF⍐)	
				3. Signal pathways (FGF-2/FGFR signaling, AMPAR-mTOR)	
stigmasterol	*Bupleurum chinense* DC. [Apiaceae; Bupleuri radix], *Angelica sinensis* (Oliv.) Diels [Apiaceae; Angelicae sinensis radix], *Codonopsis pilosula* (Franch.) Nannf. [Campanulaceae; Codonopsis radix], *Pinellia ternata* (Thunb.) Makino [Araceae; Pinelliae rhizoma], *Wurfbainia villosa* (Lour.) Skornick. and A.D.Poulsen [Zingiberaceae; Amomi fructus], *Cyperus rotundus* L. [Cyperaceae; Cyperi rhizoma], *Ziziphus jujuba* Mill. [Rhamnaceae; Jujubae fructus], *Zingiber officinale* Roscoe [Zingiberaceae; Zingiberis rhizoma recens], *Corydalis yanhusuo* (Y.H.Chou & Chun C.Hsu) W.T.Wang ex Z.Y.Su and C.Y.Wu [Papaveraceae; Corydalis rhizoma], *Gardenia jasminoides* J.Ellis [Rubiaceae; Gardeniae fructus], *Paeonia lactiflora* Pall. [Paeoniaceae; Paeoniae radix alba], *Carthamus tinctorius* L. [Asteraceae; Carthami flos], *Astragalus mongholicus* Bunge [Fabaceae; Astragali radix]	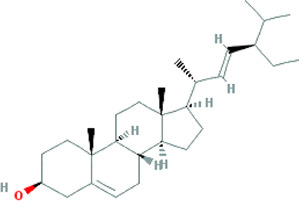	**Anxiety:** male Swiss mouse	1. Maintaining neurotransmitters homeostasis [positive modulation of GABAA receptors (GABAergic mechanism)]	Karim et al. (2021)
Isorhamnetin	*Bupleurum chinense* DC. [Apiaceae; Bupleuri radix], *Cyperus rotundus* L. [Cyperaceae; Cyperi rhizoma], *Glycyrrhiza uralensis* Fisch. ex DC. [Fabaceae; Glycyrrhizae radix et rhizoma], *Astragalus mongholicus* Bunge [Fabaceae; Astragali radix]	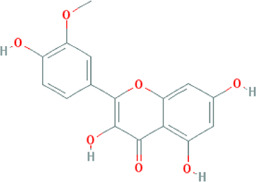	**CHD:** I/R rat H/R H9C2 TNF-*α*-HUVEC	1. Anti-damage/apoptosis (CK, LDH, Bax, cleaved-caspase-3⍗; Bcl-2⍐)	Chen et al. (2015), Zhao et al. (2018), Xu et al. (2020)
				2. Antioxidative stress (MDA⍗; SOD, CAT, GSH-Px⍐)	
				3. Anti-inflammation (ICAM-1, VCAM-1E-selectin and AP-1⍗; eNOS⍐)	
				4. Signal pathways (NF-κB; SIRT1/HO-1, Nrf2/HO-1)	
			**Depression:** starved PC12 cells	1. Inducing neuronal differentiation (NF68, NF160⍐)	Xu et al. (2012)
Baicalein	*Pinellia ternata* (Thunb.) Makino [Araceae; Pinelliae rhizoma], *Astragalus mongholicus* Bunge [Fabaceae; Astragali radix], *Paeonia lactiflora* Pall. [Paeoniaceae; Paeoniae radix alba], *Carthamus tinctorius* L. [Asteraceae; Carthami flos]	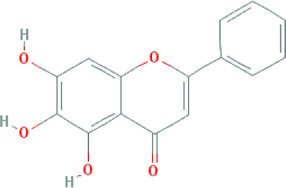	**CHD:** I/R-mouse/rat AMI rat	1. Anti-damage/apoptosis (CK, LDH, cTnI, CK-MB, Bax:Bcl-2, p53⍐)	Song et al. (2014), Kumar et al. (2016), Jie et al. (2019)
				2. Anti-inflammation (IL-1b/6, TNF-a, MCP-1, ICAM-1⍗; IL-10⍐)	
				3. Antioxidative stress (ROS, MDA, MPO⍗; CAT, SOD, GSH, GSH-PX, GSH:GSSG⍐)	
				4. Signal pathways (p38 MAPK, JNK1/2, NF-kB/p65; ERK1/2, AKT)	
			**Anxiety:** adult female Swiss mouse	1. Maintaining neurotransmitters homeostasis (dependent on GABAergic non-benzodiazepine sites but not on the 5-HT system)	de Carvalho et al. (2011)
			**Depression:** RRSD rat PDRD mouse	1. Anti-inflammation (IL-1*β*/5/6/12, IFN-*γ*⍗)	Lee et al. (2013), Zhao X. et al. (2021)
				2. Maintaining neurotransmitters homeostasis (DA, 5-HT, BDNF mRNA⍐)	
				3. Protecting synaptic plasticity (*α*-synuclein⍗; PSD95⍐)	
				4. Signal pathways (BDNF/TrkB/CREB, PI3K/Akt and CaMK II pathway)	
Tanshinone IIa	*Salvia miltiorrhiza *Bunge [Lamiaceae; Salviae miltiorrhizae radix et rhizoma]	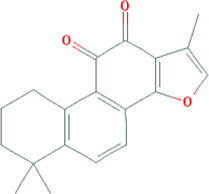	**CHD:** MI-rats/mouse I/R mouse H/R-H9C2 Ang II-CFs	1. Anti-damage/apoptosis (CK, CK-MB, LDH, Bax, cleaved-caspase-3⍗; Bcl-2⍐)	Gao S. et al. (2019), Chen L. et al. (2020), Chen et al. (2021)
				2. Anti-inflammation (IL-1*β*/6, TNF-*α*, TGF-*β*, iNOS, M1 macrophages⍗; IL-10, M2 macrophages⍐)	
				3. Antioxidative stress (superoxide anions, Nox4, MDA, ROS⍗; SOD⍐)	
				4. Antifibrosis (collagen I/III, MMP2/9, TGF-*β*, *α*-SMA⍗)	
				5. Signal pathways (lncRNA AK003290/miR-124-5p signaling)	
			**Depression:** CSRS mouse	1. Maintaining neurotransmitters homeostasis (BDNF⍐)	Lu et al. (2020)
				2. Signal pathways (ERK-CREB-BDNF pathway)	
Nobiletin	*Citrus×aurantium* L. [Rutaceae; Aurantii fructus immaturus], *Citrus×aurantium* L. [Rutaceae; Citri reticulatae pericarpium]	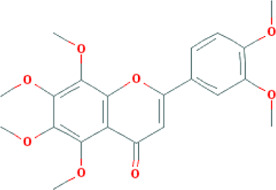	**CHD:** AMI-mouse/rat + NRVM mouse I/R rat OGD-H9C2	1. Anti-damage/apoptosis (LDH, CK-MB, Bax/Bcl2, cleaved caspase-3, caspase-12, ANP, BNP⍗)	Wu et al. (2017), Zhang B. F. et al. (2019), Liu et al. (2021), Zhou et al. (2021)
				2. Antifibrosis (*α*-SMA, collagen I/III ⍗)	
				3. Regulating autophagy (restoring autophagy flflux, lysosomes⍐)	
				4. Improving endoplasmic reticulum stress (GRP78, CHOP⍗)	
				5. Signal pathways (regulating PPAR*γ*/PGC1*α*/Nrf-2/HO-1, JNK, PI3K/AKT)	
			**Depression:** male ICR mouse LPS-depression rats + LPS + BV2 cell	1. Anti-inflammation (iNOS, IL-1*β*/6, COX2, microglial activation marker⍗)	Yi et al. (2011), Wang H. et al. (2020)
				2. Regulating autophagy (NLRP3inflammasome, ASC, caspase-1 p20⍗; LC3-II, Beclin-1⍐)	
				3. Maintaining neurotransmitters homeostasis (may interaction with the serotonergic (5-HT1A and 5-HT2 receptors), noradrenergic (*α*1-adrenoceptor) and dopaminergic (D1 and D2 receptors) systems)	
				4. Signal pathways (AMPK)	

Note: Examples of CHM in Source are derived from the results of network pharmacology analysis. ACh: acetylcholine; ACTH: adrenocorticotropic hormone; AGE: advanced glycation end product; AKT: protein kinase B; AMPAR: AMPA-type glutamate receptor; Ang II: angiotensin II; AP-1: activator protein 1; ASC: apoptosis-associated speck-like protein; ATP: adenosine triphosphate; Bax: Bcl-2 associated X protein; Bcl-2: B-cell lymphoma/leukemis-2; BDNF: brain-derived neurotrophic factor; Bim: Bcl-2-interacting mediator of cell death; BMP: bone morphogenetic protein; CaMK-II: members of the Ca(2+)/calmodulin-dependent protein kinase II; CAT: catalase; CFs: cardiac fibroblasts; CHOP: C/EBP homologous protein; CKMB: creatine kinase MB; COL1A1: Collagen 1A1; Cort: corticosterone; Cox-2: cyclooxygenase-2; CREB: cAMP response element-binding protein; CRH: corticotropin-releasing hormone; CRP: C-reactive protein; CS1: conditioned stimulus; CS2: chronic stress; CS3: citrate synthase; CSDS: chronic social defeat stress; CSRS: chronic spatial restraint stress; cTnI: cardiac troponin I; CUMS: chronic unpredictable mild stress; DA: dopamine; DM: diabetes mellitus; eNOS: endothelial nitric oxide synthase; ERK: extracellular signal-regulated kinase; FAAH: fatty-acid amide hydrolase; FGF-2: fibroblast growth factor 2; FGFR: fibroblast growth factor receptor; FXR: frnesoid X receptor; GABA: gamma-aminobutyric acid; GRP78: Glucose-Regulated Protein 78; GSH: glutathione; GSH-Px: glutathione peroxidase; GSK-3*β*: glycogen synthase kinase-3*β*; GST: glutathione-s transferase; GSSG: glutathione disulfide; HNC: hippocampal neuron cell; HO-1: heme oxygenase-1; 5-HT: 5-hydroxytryptamine; HUVEC: human umbilical vein endothelial cells; ICAM-1: intercellular adhesion molecule-1; IFN: interferon; IL: interleukin; iNOS: inducible nitric oxide synthase; I/R: ischemia reperfusion; JNK: Jun N-terminal kinase; LC3: light chain 3; LDH: lactate dehydrogenase; Lp-PLA2: lipoprotein-associated Phospholipase A2; LVEDVi: left ventricular end-diastolic volumes index; LVEF: left ventricular ejection fraction; LVESVi: left ventricular end-systolic volume index; MAO: monoamine oxidase; MAPK: mitogen-activated protein kinase; MCP-1: monocyte chemoattractant protein-1; MDA: malondialdehyde; MI: myocardial infarction; MMP: matrix metalloproteinases; MPO: myeloperoxidase; Mst1:macrophage stimulating 1; mTBI: mild traumatic brain injury; mTOR: mammalian target of rapamycin; mtPTP: mitochondrial permeability transition pore; NE: noradrenaline; NF: neurofilaments; NF-κB: Nuclear factor-kappaB; NLRP3: NACHT, LRR, and PYD domains-containing protein 3; Nox4: NADPH oxidase 4; Nrf2: Nuclear factor E2-related factor 2; NRVM: neonatal rat ventricular myocyte; OGD: oxygen-glucose deprivation model; OID: ovariectomy-induced depression primary; ox-LDL: oxidized low-density lipoprotein; PDRD: Parkinson’s disease-related depression; PGC1*α*: peroxisome proliferator-activated receptor-*γ* co-activator-1*α*; PI3K: phosphatidylinositol 3-kinase; PMAT: plasma membrane monoamine transporter; PPAR: peroxisome proliferator-activated receptor; PRX: Peroxiredoxins; PSD95: postsynaptic density 95; RAGE: receptor for AGE; ROS: reactive oxygen species; RRSD: repeated restraint stress-induced depression-like behavior; SIA: stress-induced anxiety; SERCA2a:sarcoplasmic/endoplasmic reticulum Ca2+ ATPase 2a; SOD: superoxide dismutase; Sp1: specificity protein 1; STAT3: signal transducers and activators of transcription3; TC: total cholesterol; TG: triglyceride; TGF-*β*: transforming growth factor-*β*; TNF-*α*: tumor necrosis factor-*α*; TrkB: tropomycin receptor kinase B; TUNEL: terminal dUTP nick-end labeling; US: unconditioned stimulus; VCAM-1: vascular cell adhesion molecule-1; *α*-SMA: *α*-smooth muscle actin.

### Potential Relevant Mechanisms

The effects and mechanisms of the primary active compounds (top 10) were searched in the Web of Science database. As shown in Table 3, the experimental research of quercetin, kaempferol, luteolin, beta-sitosterol, puerarin, and baicalein covered CHD, anxiety, and depression. Models of myocardial infarction or ischemia reperfusion were commonly used in the study of CHD, while the ICR mice and multiple stress-stimulated rats were selected for anxiety and depression research.

The related mechanisms of these top 10 active ingredients in CHD, anxiety, and depression are summarized in Table 3, which mainly includes anti damage/apoptosis, anti-inflammation, antioxidative stress, antifibrosis, maintaining neurotransmitters homeostasis, and regulating autophagy. In addition, myocardial injure biomarkers (lactate dehydrogenase, creatine kinase MB, cardiac troponin I) and the damage/apoptosis biomarkers (Bcl-2 associated X protein, cleaved caspase-3, p53, B-cell lymphoma/leukemis-2) could be regulated by quercetin, kaempferol, luteolin, beta-sitosterol, puerarin, isorhamnetin, baicalein, tanshinone IIa, and nobiletin. These phytochemicals were also reported to exert an anti-inflammatory effect by reducing the levels of interleukin (IL) -1*β*/6, tumor necrosis factor-*α*, vascular cell adhesion molecule-1, intercellular adhesion molecule-1, E-selectin, or elevating the IL-10 level in CHD patients. The anti-inflammatory role of quercetin, puerarin, or nobiletin was reported in anxiety or depression. Additionally, almost all active ingredients except stigmasterol and nobiletin possessed the functions of antioxidative stress and balancing level of reactive oxygen species, malondialdehyde, myeloperoxidase, and catalase, superoxide dismutase, glutathione. Quercetin, tanshinone IIa, and nobiletin were reported to reduce the levels of *α*-smooth muscle actin, angiotensin II, collagen I/III, matrix metalloproteinases 2/9, transforming growth factor (TGF)-*β*, and Smad7 to prevent myocardial fibrosis, which was one of the complications associated with myocardial infarction. Besides, the imbalance of adrenocorticotropic hormone, 5-hydroxytryptamine, brain-derived neurotrophic factor (BDNF), acetylcholine, noradrenaline, dopamine, and gamma-aminobutyric acid, which caused anxiety or depression, could be regulated by quercetin, kaempferol, luteolin, beta-sitosterol, puerarin, stigmasterol, baicalein, tanshinone IIa, or nobiletin. Isorhamnetin and baicalein could improve the depression by inducing neuronal differentiation and protecting synaptic plasticity, respectively. The roles of active compounds in regulating autophagy and improving mitochondria were also reported. Overall, the related mechanisms of TCM-active compounds in treating CHD with anxiety or depression contained a variety of signaling pathways, such as nuclear factor-kappa B, mitogen-activated protein kinase, Jun N-terminal kinase, extracellular signal-regulated kinase1/2, signal transducers and activators of transcription3, TGF-*β*1/Smad3, phosphatidylinositol 3-kinase/protein kinase B, and BDNF.

## Discussion

There is accumulating evidence showing high prevalence of anxiety and depression comorbidities in patients with CHD. SSRIs and benzodiazepines are frequently used for treating depression or anxiety disorders, and the effectiveness of these drugs on psychiatric disorders has also been acknowledged (Davies et al., 2004). However, the side effects, such as suicidal ideation, sexual dysfunction, and dependency, have not been resolved (Lakhan and Vieira, 2010; Ko et al., 2020). In addition, it is a common clinical phenomenon that CHD patients show subsyndromal anxiety or depression-like symptoms that do not meet the diagnostic criteria of anxiety or depression (Cohen et al., 2006; Kasckow et al., 2013). The issue of treatment for these patients still deserves much attention.

TCM has been reported to be effective in treating CHD, anxiety, and depression with a less adverse effect, and might be a potential therapeutic option for patients with subsyndromal anxiety or depression. However, the efficacy and benefit of CHM in treating CHD with anxiety or depression still need to be further verified due to poor methodological quality and potential confounding factors. This meta-analysis and systematic review was performed to provide the evidence for the application of CHM in CHD patients with anxiety or depression. Thirty-two studies (15 CHD with anxiety, and 17 CHD with depression) were included for the evaluation of the efficacy of CHM. The results showed that CHM had a significant benefit on anxiety and depression in CHD patients, and its efficacy was not inferior to that of WM. Importantly, CHM also had a significant advantage to alleviate the angina symptom compared with blank control and WM groups. Besides that, there were no obvious adverse effects of CHM in the included studies (Zhang et al., 2012; Qin and Liu, 2013; Zhu, 2013; Mo et al., 2016; Shi et al., 2016; Guo, 2017; Wang Y. et al., 2018; Wang C., 2018; Wang D. D., 2018; Chen, 2019; Lu, 2019; Yang, 2019; Huang et al., 2020).

Furthermore, the frequency of CHM used in the included studies was analyzed, and the commonly used drugs were analyzed by network pharmacology. The results concluded that the CHM regulating Qi and promoting blood circulation, including *Bupleurum chinense* DC. [Apiaceae; Bupleuri radix], *Glycyrrhiza uralensis* Fisch. ex DC. [Fabaceae; Glycyrrhizae radix et rhizoma], *Ligusticum chuanxiong* Hort. [Apiaceae; Chuanxiong rhizoma], *Salvia miltiorrhiza* Bunge [Lamiaceae; Salviae miltiorrhizae radix et rhizoma], *Angelica sinensis* (Oliv.) Diels [Apiaceae; Angelicae sinensis radix], *Paeonia lactiflora* Pall. [Paeoniaceae; Paeoniae radix alba], *Pinellia ternata* (Thunb.) Makino [Araceae; Pinelliae rhizoma], *Curcuma aromatica* Salisb. [Zingiberaceae; Curcumae radix], and *Citrus×aurantium* L. [Rutaceae; Aurantii fructus] were commonly used for CHD with anxiety and depression. The phytochemicals identified in the CHM could act on the pathological targets of CHD, anxiety, and depression simultaneously.

Inflammatory response to vascular injury participates in the pathological processes of the atherosclerosis and CHD, and is associated with the increased risk of cardiovascular events and recurrent myocardial infarction (Fioranelli et al., 2018; Zhang K. J. et al., 2019). Oxidative stress is also an important factor involved in myocardial cell injury and apoptosis caused by ischemia reperfusion, which is followed by heart failure and myocardial fibrosis (Li et al., 2018; Yang et al., 2019). The effectiveness of CHM ingredients including quercetin, kaempferol, luteolin, beta-sitosterol, puerarin, and baicalein was reported in the experimental research on CHD. Puerarin, quercetin, and tanshinone IIa were also shown to have a satisfactory efficacy in improving clinical prognosis (Mao et al., 2019; Zhang S. et al., 2019; Dehghani et al., 2021). Additionally, inflammation and oxidative stress could cause neuron damage and neurotransmitter disorder, leading to anxiety and depression (Bankier et al., 2009; Liu et al., 2015; van Dooren et al., 2016; Salim, 2017; Wang Y. L. et al., 2018). Quercetin, kaempferol, luteolin, and puerarin also showed a positive effect in curing anxiety and depression.

Overall, these findings reveal that the CHM has a satisfactory efficacy for CHD with anxiety and depression, especially for improving the symptom of angina pectoris. Of importance, CHM itself contains multiple components that play critical functions in a large number of signaling pathways involved in distinct biological processes of CHD with anxiety or depression mainly including anti-damage/apoptosis, anti-inflammation, antioxidative stress, and maintaining neurotransmitters homeostasis. Compared with WM’s single effect on the nervous system, CHM may extert its functions in multiple places and systems by targeting distinct factors in CHD with anxiety or depression to improve both CHD and anxiety/depression syndromes.

## Limitations

First, the sample size in each group of included studies was not more than 50, except the study by Wang Y. L. et al. (2018), and the sample size needs to be expanded in future studies. Second, it is difficult to perform double blind due to the special smell and taste of TCM decoction. Also, the characteristics of TCM treatment affect the implementation of double blind. Additionally, the blinding of outcome assessment was conducted in 2 of 32 studies (Qi and Song, 2017; Zhang and Jin, 2021). Therefore, the strict trial design is also necessary to further verify the efficacy of CHM.

## Conclusion

CHM had a significant efficacy for the treatment of CHD patients with anxiety or depression. Particularly, CHM could improve the symptoms of angina pectoris while alleviating anxiety and depression. The main mechanisms underlying the functions of these CHM-active ingredients might involve anti-damage/apoptosis, anti-inflammation, antioxidative stress, antifibrosis, maintaining neurotransmitters homeostasis, and regulating autophagy.

## Data Availability

The original contributions presented in the study are included in the article/Supplementary Material, further inquiries can be directed to the corresponding authors.

## Abbreviations

AF, angina frequency; AS, angina stability; CHD, coronary heart disease; CHM, Chinese herbal medicine; ECG, electrocardiogram; HAMA, Hamilton anxiety scale; HAMD, Hamilton depression scale; TCM, traditional Chinese medicine; WM, Western medicine.
