# Supplementary material for: Evidence and Characteristics of Traditional Chinese Medicine for Coronary Heart Disease Patients With Anxiety or Depression: A Meta-Analysis and Systematic Review
Source: Front Pharmacol. 2022 May 5;13:854292. doi: 10.3389/fphar.2022.854292 (PMC9117623; doi:10.3389/fphar.2022.854292)
Supplement: Supplementary file 1 [file DataSheet1.PDF]

Searching strategy of English database (PubMed, Web of science, Embase, Cochrane)
#1: (Coronary Disease OR Coronary Diseases OR Disease, Coronary OR Diseases, Coronary OR Coronary Heart Disease OR Coronary Heart Diseases OR Disease, Coronary Heart OR Diseases, Coronary Heart OR Heart Disease, Coronary OR Heart Diseases, Coronary)
#2: (coronary artery disease OR Artery Disease, Coronary OR Artery Diseases, Coronary OR Coronary Artery Diseases OR Left Main Coronary Artery Disease OR Left Main Disease OR Left Main Diseases OR Left Main Coronary Disease OR Coronary Arteriosclerosis OR Arterioscleroses, Coronary OR Coronary Arterioscleroses OR Atherosclerosis, Coronary OR Atheroscleroses, Coronary OR Coronary Atheroscleroses OR Coronary Atherosclerosis OR Arteriosclerosis, Coronary)
#3: (Myocardial Infarction OR Infarction, Myocardial OR Infarctions, Myocardial OR Myocardial Infarctions OR Cardiovascular Stroke OR Cardiovascular Strokes OR Stroke, Cardiovascular OR Strokes, Cardiovascular OR Myocardial Infarct OR Infarct, Myocardial OR Infarcts, Myocardial OR Myocardial Infarcts OR Heart Attack OR Heart Attacks)
#4: (Acute Coronary Syndrome OR Acute Coronary Syndromes OR Coronary Syndrome, Acute OR Coronary Syndromes, Acute OR Syndrome, Acute Coronary OR Syndromes, Acute Coronary)
#5: (Depression OR Depressions OR Depressive Symptoms OR Depressive Symptom OR Symptom, Depressive OR Symptoms, Depressive OR Emotional Depression OR Depression, Emotional OR Depressions, Emotional OR Emotional Depressions)
#6: (Depressive Disorder OR Depressive Disorders OR Disorder, Depressive OR Disorders, Depressive OR Neurosis, Depressive OR Depressive Neuroses OR Depressive Neurosis OR Neuroses, Depressive OR Depression, Endogenous OR Depressions, Endogenous OR Endogenous Depression OR Endogenous Depressions OR Depressive Syndrome OR Depressive Syndromes OR Syndrome, Depressive OR Syndromes, Depressive OR Depression, Neurotic OR Depressions, Neurotic OR Neurotic Depression OR Neurotic Depressions OR Melancholia OR Melancholias OR Unipolar Depression OR Depression, Unipolar OR Depressions, Unipolar OR Unipolar Depressions)
#7: (anxiety OR Angst OR Nervousness OR Hypervigilance OR Anxiousness OR Social Anxiety OR Anxieties, Social OR Anxiety, Social OR Social Anxieties)
#8: (((#1) OR #2) OR #3) OR #4
#9: ((#5) OR #6) OR #7
#10: (#8) AND #9

#11: (Medicine, Chinese Traditional OR Traditional Chinese Medicine OR Traditional Medicine, Chinese OR Zhong Yi Xue OR Chinese Traditional Medicine OR Chinese Medicine, Traditional)
#12: (herbal medicine OR Medicine, Herbal OR herb OR Chinese herbal medicine)
#13: (#11) OR #12

#14: (Randomized controlled trail OR Randomized OR Placebo)
#15: ((#10) AND #13) AND #14

Searching strategy of Chinese database (China National Knowledge Infrastructure, WanFang Database, VIP Database and SinoMed)
（（（“冠心病”OR“冠状动脉粥样硬化心脏病”OR“心梗”OR“急性冠脉综合征”OR“心肌梗死”OR“急性冠脉综合症”OR“急性冠状动脉综合征”OR“急性冠状动脉综合症”）AND（“焦虑”OR“抑郁”OR“情志”OR“情绪”））AND（“中医”OR“中药”OR“中医药”OR“草药”OR“中草药”OR“方剂”OR“药方”OR“颗粒”OR“中成药”））AND（“随机对照试验”OR“随机对照研究”OR“RCT”OR“随机”）

Table S1a. The quality assessment of CHD with anxiety.

Study(year)	A	B	C	D	E	F	G
Mo 2016	L	L	L	U	L	L	U
Guo 2017	L	U	H	U	L	L	U
Li 2017	L	U	H	U	L	L	U
Qi 2017	L	U	H	L	L	L	U
Zhang 2017	L	U	H	U	L	L	U
Qin 2018	L	U	H	U	L	L	U
Wang 2018	L	L	L	U	L	L	U
Chen 2019	L	U	H	U	L	L	U
Dong 2019	L	U	H	U	L	L	U
Yang 2019	U	U	H	U	L	L	U
Zhang 2019	L	U	H	U	L	L	U
Zhao 2019	L	U	H	U	L	L	U
Jin 2021	L	U	H	U	L	L	U
Wang 2021	L	U	H	U	L	L	U
Zhang 2021	L	U	H	L	L	L	U

Table S1b. The quality assessment of CHD with depression.

Study(year)	A	B	C	D	E	F	G
Sun 2011	L	L	H	U	L	L	U
Lin 2012	U	U	L	U	L	L	U
Zhang 2012	L	U	H	U	L	L	U
Qin 2013	U	U	H	U	L	L	U
Zhu 2013	L	U	H	U	L	L	U
Gu 2014	L	U	H	U	L	L	U
Shang 2014	L	U	H	U	L	L	U
Mu 2015	U	U	H	U	L	L	U
Shi 2016	U	U	H	U	L	L	U
Li 2017	L	U	H	U	L	L	U
Su 2017	U	U	L	U	L	L	U
Wang1 2018	L	U	H	U	L	L	U
Wang2 2018	L	U	H	U	L	L	U
Shi 2018	U	U	H	U	L	L	U
Lu 2019	U	U	H	U	L	L	U
Huang 2020	L	U	H	U	L	L	U
Zhang 2020	L	U	H	U	L	L	U

A: adequate sequence generation; B: concealment of allocation; C: blinding of participants; D: blinding of outcome assessment; E: incomplete out-come data; F: selective reporting; G: other bias; L: low risk; U: unclear risk, the information was inadequate in the text.

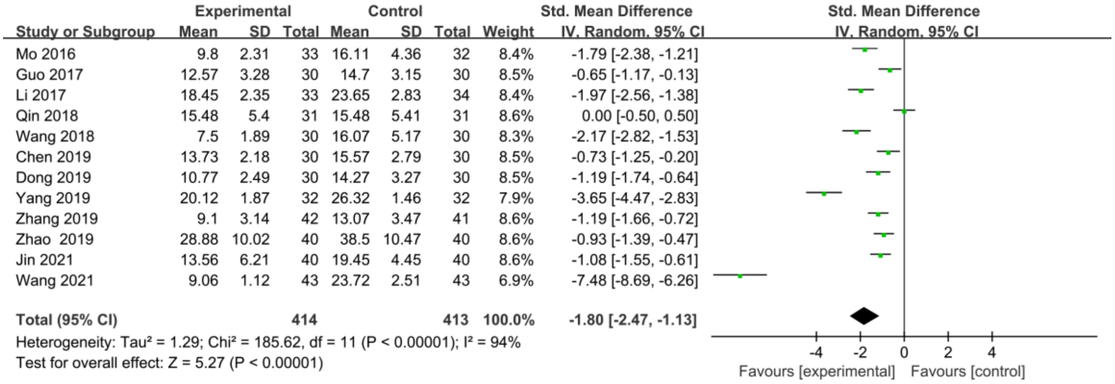

Figure S1. Forest plot: the post-treatment score of HAMA of CHM for CHD with anxiety compared with control groups.

Table S2. The frequency statistics of CHM in included studies

The CHM of CHD with anxiety			The CHM of CHD with depression		
**Chinese name**	**Standardized name**	**Frequency**	**Chinese name**	**Standardized name**	**Frequency**
Chaihu	*Bupleurum chinense* DC. [Apiaceae; Bupleuri radix]	11	Chaihu	*Bupleurum chinense* DC. [Apiaceae; Bupleuri radix]	12
Gancao	*Glycyrrhiza uralensis Fisch. ex* DC. [Fabaceae; Glycyrrhizae radix et rhizoma]	8	Danshen	*Salvia miltiorrhiza* Bunge [Lamiaceae; Salviae miltiorrhizae radix et rhizoma]	11
Zhike	*Citrus×aurantium* L. [Rutaceae; Aurantii fructus]	7	Gancao	*Glycyrrhiza uralensis Fisch. ex* DC. [Fabaceae; Glycyrrhizae radix et rhizoma]	11
Chuanxiong	*Ligusticum chuanxiong* Hort. [Apiaceae; Chuanxiong rhizoma]	6	Yujin	*Curcuma aromatica* Salisb. [Zingiberaceae; Curcumae radix]	10
Danggui	*Angelica sinensis* (Oliv.) Diels [Apiaceae; Angelicae sinensis radix]	5	Baishao	*Paeonia lactiflora* Pall. [Paeoniaceae; Paeoniae radix alba]	8
Dangshen	*Codonopsis pilosula* (Franch.) Nannf. [Campanulaceae; Codonopsis radix]	5	Chuanxiong	*Ligusticum chuanxiong* Hort. [Apiaceae; Chuanxiong rhizoma]	8
Baishao	*Paeonia lactiflora* Pall. [Paeoniaceae; Paeoniae radix alba]	4	Danggui	*Angelica sinensis* (Oliv.) Diels [Apiaceae; Angelicae sinensis radix]	8
Banxia	*Pinellia ternata* (Thunb.) Makino [Araceae; Pinelliae rhizoma]	4	Fuling	*Poria cocos* (Schw.) Wolf [Polyporaceae; Poria]	7
Guizhi	*Neolitsea cassia* (L.) Kosterm. [Lauraceae; Cinnamomi ramulus]	4	Suanzaoren	*Ziziphus jujuba* Mill. [Rhamnaceae; Ziziphi spinosae semen]	7
Xiangfu	*Cyperus rotundus* L. [Cyperaceae; Cyperi rhizoma]	4	Banxia	*Pinellia ternata* (Thunb.) Makino [Araceae; Pinelliae rhizoma]	6

Dazao	*Ziziphus jujuba* Mill. [Rhamnaceae; Jujubae fructus]	3	Hehuanpi	*Albizia julibrissin* Durazz. [Fabaceae; Albiziae flos]	6
Danshen	*Salvia miltiorrhiza* Bunge [Lamiaceae; Salviae miltiorrhizae radix et rhizoma]	3	Sharen	*Wurfbainia villosa* (Lour.) Skornick. & A.D.Poulsen [Zingiberaceae; Amomi fructus]	6
Fuling	*Poria cocos* (Schw.) Wolf [Polyporaceae; Poria]	3	Chenpi	*Citrus × aurantium* L. [Rutaceae; Citri reticulatae pericarpium]	5
Huangqi	*Astragalus mongholicus* Bunge [Fabaceae; Astragali radix]	3	Zhike	*Citrus×aurantium* L. [Rutaceae; Aurantii fructus]	5
Jiegen	*Platycodon grandiflorus* (Jacq.) A.DC. [Campanulaceae; Platycodonis radix]	3	Huangqi	*Astragalus mongholicus* Bunge [Fabaceae; Astragali radix]	4
Longgu	*Os Draconis*	3	Shengjiang	*Zingiber officinale* Roscoe [Zingiberaceae; Zingiberis rhizoma recens]	4
Muli	*Ostrea gigas* Thunberg [Ostreidae; Ostreae concha]	3	Wuweizi	*Schisandra chinensis* (Turcz.) Baill. [Schisandraceae; Schisandrae chinensis fructus]	4
Shengjiang	*Zingiber officinale* Roscoe [Zingiberaceae; Zingiberis rhizoma recens]	3	Xiangfu	*Cyperus rotundus* L. [Cyperaceae; Cyperi rhizoma]	4
Yujin	*Curcuma aromatica* Salisb. [Zingiberaceae; Curcumae radix]	3	Yanhusuo	*Corydalis yanhusuo* (Y.H.Chou & Chun C.Hsu) W.T.Wang ex Z.Y.Su & C.Y.Wu [Papaveraceae; Corydalis rhizoma]	4
Yanhusuo	*Corydalis yanhusuo* (Y.H.Chou & Chun C.Hsu) W.T.Wang ex Z.Y.Su & C.Y.Wu [Papaveraceae; Corydalis rhizoma]	3	Yuanzhi	*Polygala tenuifolia* Willd. [Polygalaceae; Polygalae radix]	4
Zhizi	*Gardenia jasminoides* J.Ellis [Rubiaceae; Gardeniae fructus]	3	Baihe	*Lilium lancifolium* Thunb. [Liliaceae; Lilii bulbus]	3
			Chishao	*Paeonia anomala subsp. veitchii* (Lynch) D.Y.Hong & K.Y.Pan [Paeoniaceae; Paeoniae radix rubra]	3
			Dazao	*Ziziphus jujuba* Mill. [Rhamnaceae; Jujubae fructus]	3
			Gualou	*Trichosanthes kirilowii* Maxim. [Cucurbitaceae; Trichosanthis fructus]	3
			Guizhi	*Neolitsea cassia* (L.) Kosterm. [Lauraceae; Cinnamomi ramulus]	3
			Hehuanhua	*Albizia julibrissin* Durazz. [Fabaceae; Albiziae flos]	3

	Honghua	*Carthamus tinctorius* L. [Asteraceae; Carthami flos]	3
	Huangqin	*Scutellaria baicalensis* Georgi [Lamiaceae; Scutellariae radix]	3
	Shichangpu	*Acorus calamus var. angustatus* Besser [Acoraceae; Acori tatarinowii rhizoma]	3
	Zhizi	*Gardenia jasminoides* J.Ellis [Rubiaceae; Gardeniae fructus]	3

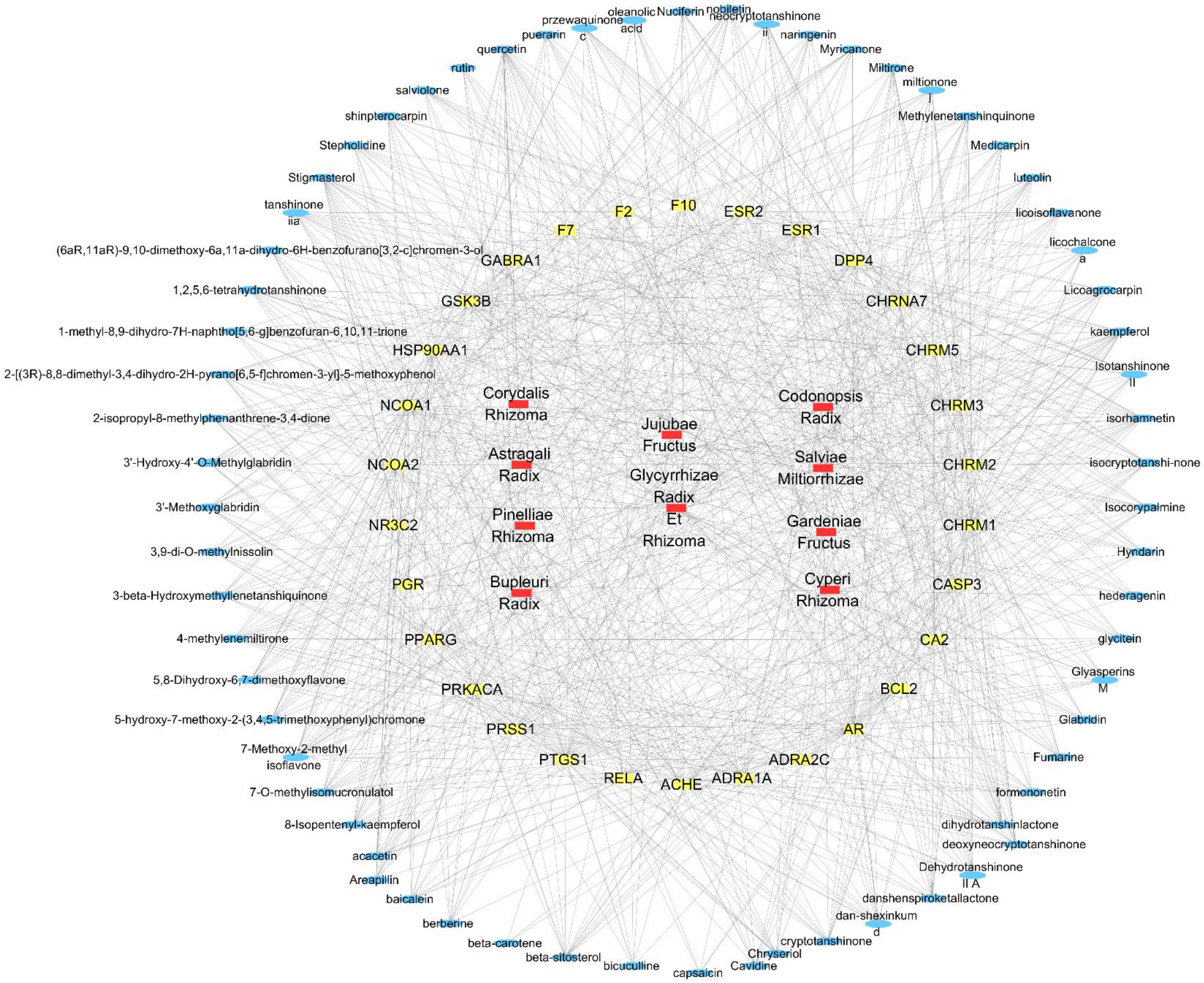

Figure S2a. The networks of active ingredients-disease targets of CHD with anxiety. Red: CHM; yellow: disease targets; blue: active ingredients (the degree centrality was more than the average degree).

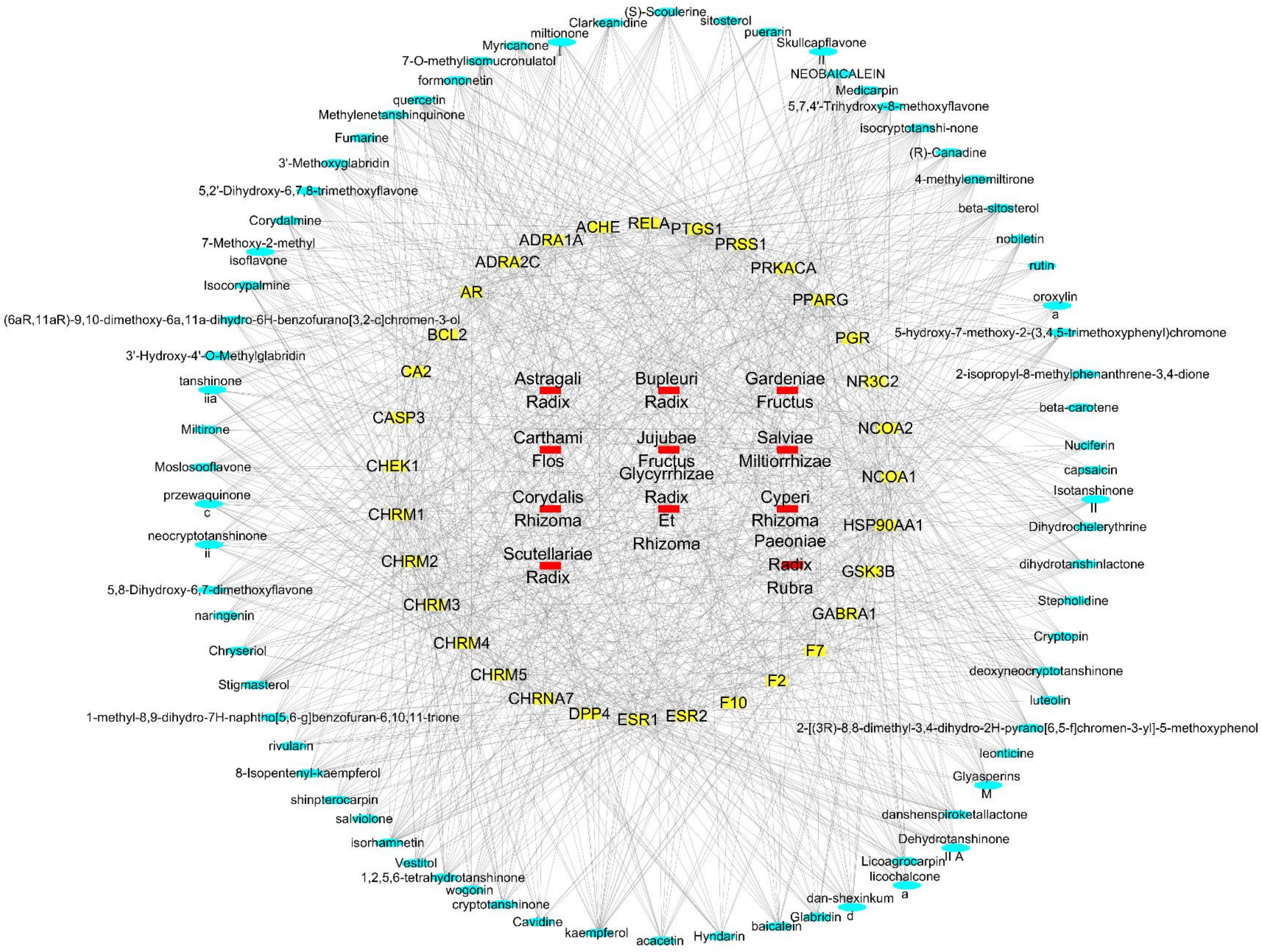

Figure S2b. The networks of active ingredients-disease targets of CHD with depression. Red: CHM; yellow: disease targets; blue: active ingredients (the degree centrality was more than the average degree).
